# A Species-Specific *COI* PCR Approach for Discriminating Co-Occurring Thrips Species Using Crude DNA Extracts

**DOI:** 10.3390/biology15020171

**Published:** 2026-01-17

**Authors:** Qingxuan Qiao, Yaqiong Chen, Jing Chen, Ting Chen, Huiting Feng, Yussuf Mohamed Salum, Han Wang, Lu Tang, Hongrui Zhang, Zheng Chen, Tao Lin, Hui Wei, Weiyi He

**Affiliations:** 1 Fujian Engineering Research Center for Green Pest Management, Fujian Key Laboratory for Monitoring and Integrated Management of Crop Pests, Institute of Plant Protection, Fujian Academy of Agricultural Sciences, Fuzhou 350013, China; qqx17862665489@163.com (Q.Q.); chen_yaqiong@126.com (Y.C.); ct318815@163.com (T.C.); chenzheng@faas.cn (Z.C.); maludongzuo@163.com (T.L.); 2 State Key Laboratory of Agricultural and Forestry Biosecurity, Institute of Applied Ecology, Fujian Agriculture and Forestry University, Fuzhou 350002, China; cj13276011790@163.com (J.C.); feng06092022@126.com (H.F.); 13919892472@163.com (H.W.); tlttll2003@163.com (L.T.); 3 Plant Protection College, Yunnan Agricultural University, Kunming 650201, China; hongruizh@126.com

**Keywords:** *COI* gene, species-specific primers, PBS-based DNA extraction

## Abstract

Thrips are cosmopolitan agricultural pests and important vectors of plant viruses, and the increasing coexistence of multiple morphologically similar species has intensified the demand for species-specific molecular identification. However, traditional morphological identification and PCR assays using universal primers are often inadequate for mixed-species samples and field-adaptable application. In this study, we developed a species-specific molecular identification framework targeting a polymorphism-rich region of the mitochondrial cytochrome c oxidase subunit I (*COI*) gene, which is more time-efficient than sequencing-based *COI* DNA barcoding, for four economically important thrips species in southern China, including the globally invasive *Frankliniella occidentalis*. By aligning *COI* sequences, polymorphism-rich regions were identified and used to design four species-specific primer pairs, each containing a diagnostic 3′-terminal nucleotide. These primers were combined with a PBS-based DNA extraction workflow optimized for single-insect samples that minimizes dependence on column-based purification. The assay achieved a practical detection limit of 1 ng per reaction, demonstrated species-specific amplification, and maintained reproducible amplification at DNA inputs of ≥1 ng per reaction. Notably, PCR inhibition caused by crude extracts was effectively alleviated by fivefold dilution. Although the chemical identities of the inhibitors remain unknown, interspecific variation in inhibition strength was observed, with *T. hawaiiensis* exhibiting the strongest suppression, possibly due to differences in lysate composition. This integrated framework balances target specificity, operational simplicity, and dilution-mitigated inhibition, providing a field-adaptable tool for thrips species identification and invasive species monitoring. Moreover, it provides a species-specific molecular foundation for downstream integration with visual nucleic acid detection platforms, such as the CRISPR/Cas12a system, thereby facilitating the future development of portable molecular identification workflows for small agricultural pests.

## Simple Summary

Thrips are tiny insects that feed on many crops and can spread plant viruses. Different thrips species often co-occur on the same host plants but are morphologically similar, making species-level molecular identification challenging under field-oriented conditions. This is important because species differ in how much damage they cause; how they respond to insecticides; and whether they spread viruses. In this study, we developed a simple detection system that can quickly tell apart four important thrips species that attack crops in southern China; including one globally invasive species; the western flower thrips. The test uses species-diagnostic regions of the mitochondrial *COI* gene and a short-turnaround DNA extraction workflow for processing individual insects. Our results show that species-specific and reproducible amplification can be achieved under mixed-species backgrounds, and that PCR inhibition can be effectively alleviated by mild dilution of crude insect lysates. This framework supports laboratory- and field-oriented molecular identification workflows for greenhouse and open-field thrips surveillance and provides a methodological basis for potential integration with simplified visual nucleic acid readout platforms in future applications.

## 1. Introduction

Polyphagous thrips are globally important agricultural pests widely distributed across tropical and subtropical regions. Their minute body size and high reproductive capacity enable infestation across diverse crops [1,2]. Thrips possess piercing-sucking mouthparts and initiate feeding by rasping plant surfaces before inserting their stylets to extract cellular contents. This feeding behavior induces tissue necrosis, leaf deformation, and fruit developmental abnormalities, ultimately compromising both crop yield and quality [3,4]. Furthermore, several species function as competent vectors of plant viruses such as tomato spotted wilt virus (TSWV), impatiens necrotic spot virus (INSV), and groundnut ringspot virus (GRSV), thereby posing a dual threat through both direct feeding damage and virus transmission [5,6]. Together, these traits render thrips a persistent threat to the biosecurity and sustainability of modern agricultural production.

In protected cropping systems, thrips exhibit continuous year-round occurrence and rapid generational turnover [7,8]. Their capacity for parthenogenetic reproduction, rapid evolution of insecticide resistance, and pronounced cryptic behavior further contributes to their persistence in high-value horticultural crops [8,9]. In southern coastal regions of China, the expansion of protected cultivation and intensified cross-regional trade have markedly reshaped the regional distribution patterns and dominant species composition of thrips populations [10,11]. Currently, the flower thrips *Frankliniella intonsa* (Trybom, 1895) (Thysanoptera: Thripidae), the bean thrips *Megalurothrips usitatus* (Bagnall, 1913), and the Hawaiian flower thrips *Thrips hawaiiensis* (Morgan, 1913) dominate local agricultural systems in Fujian Province [12,13], whereas the invasive western flower thrips *Frankliniella occidentalis* (Pergande, 1895), originally absent from most provinces in China, has in recent years rapidly expanded its distribution range, progressively establishing stable populations and exhibiting strong competitive displacement of native species, thereby emerging as a dominant pest in greenhouse-based protected cropping systems [14].

As a result, agricultural ecosystems increasingly exhibit the coexistence of multiple thrips species, with interspecific resource partitioning and competitive interactions that may influence population dynamics [15,16]. Accurate identification and real-time monitoring of dominant thrips populations are therefore fundamental for elucidating population succession and coexistence mechanisms and serve as essential prerequisites for precision pest management [17,18]. However, the high morphological similarity among species, fragility of specimens, and frequent cooccurrence substantially limit the accuracy and efficiency of traditional morphological approaches, particularly under field monitoring conditions [19,20]. Although mitochondrial cytochrome c oxidase subunit I (*COI*) markers have been widely applied in molecular identification, barcoding approaches rely on sequencing platforms and thus fall short of supporting rapid, field-adaptable diagnostics [21]. Despite the broad adoption for amplifying conserved mitochondrial barcoding loci, universal *COI* primers are not intended for direct species discrimination at the PCR level. When applied to samples containing closely related or co-occurring thrips species, universal primers may yield mixed amplification products, which require subsequent sequencing for accurate species assignment and thus limit their suitability for rapid, field-adaptable identification without sequencing [22]. The limitations are further amplified by the practical constraints of current molecular workflows, which usually depend on specialized laboratory equipment and controlled laboratory conditions. These include thermocyclers, centrifuges, and gel-based detection systems, as well as multi-step DNA purification procedure, that involve multiple handling and transfer steps, even in commonly used column-based extraction workflows, thereby limiting the applicability to low-input analyses from individual insects under field conditions [23]. Compounding this challenge, crude lysates from individual insects may contain co-extracted substances that interfere with downstream PCR amplification, thereby reducing amplification efficiency and reliability rather than the efficiency of DNA extraction itself [24,25].

In summary, the frequent coexistence of multiple thrips species and dynamic shifts in species dominance underscores the pressing need for a molecular species identification framework that integrates species-level resolution, operational simplicity, and field-adaptable applicability. To address this challenge, the present study focused on thrips species most frequently encountered in local agricultural systems, together with a globally invasive species *F. occidentalis*, and performed a systematic assessment of mitochondrial *COI* polymorphisms to pinpoint hypervariable regions conducive to species-specific primer development. Concurrently, six DNA extraction protocols were evaluated and optimized to establish a short-turnaround, species-specific molecular identification that reconciles species-level resolution with field-compatible applicability and is more time-efficient than sequencing-based barcoding. Departing from conventional *COI* barcoding approaches restricted to laboratory-based sequencing, this study combines polymorphism-guided primer design with a PBS-based DNA extraction strategy for single-insect samples that requires no column-based purification steps, establishing a modular identification framework oriented toward laboratory and field applications. Such integration effectively bridges the gap between laboratory molecular assays and field-adaptable surveillance and lays a molecular foundation for seamless incorporation of isothermal amplification and visual nucleic acid detection platforms, including CRISPR/Cas12a-based assays, to advance intelligent pest monitoring and to accelerate the transition toward portable, data-driven molecular species identification frameworks in smart agriculture.

## 2. Materials and Methods

### 2.1. Insect Materials and Rearing Maintenance

Four thrips species used in this study were *F. intonsa*, *F. occidentalis*, *M. usitatus*, and *T. hawaiiensis*. Among them, *F. intonsa*, *M. usitatus*, and *T. hawaiiensis* are dominant native species commonly found in agricultural systems throughout Fujian Province, while *F. occidentalis* has rapidly spread in China. The *F. occidentalis* laboratory strain was established in 2018 from a starter population provided by the Institute of Biotechnology and Germplasm Resources, Yunnan Academy of Agricultural Sciences. Field-derived strains of *F. intonsa* (2021, collected on cucumber, *Cucumis sativus* L., 1753), *M. usitatus* (2021, collected on cowpea, *Vigna unguiculata* (L.) Walp., 1843), and *T. hawaiiensis* (2017, collected on sweet osmanthus, *Osmanthus fragrans* (Thunb.) Lour., 1790) were established independently from populations sampled in Minhou, Fuzhou, Fujian, China. All four species have been maintained under laboratory conditions for multiple years. Each species has been reared independently under strict biosecurity protocols to prevent cross-contamination and to ensure stable laboratory populations. Species identity was regularly verified using morphological characteristics and mitochondrial *COI* barcoding. All species were maintained in a controlled-environment chamber (CRX-350B, Fujian Zhongyouling Technology Co., Ltd., Fuzhou, China) set at 22 ± 1 °C, 75 ± 5% RH, and a 16:8 h light:dark (L:D) photoperiod. Strain health and developmental synchrony were routinely monitored throughout rearing.

Rearing followed a previously described system [26], which was further optimized in this study to enhance egg collection efficiency and minimize contamination risk. For *F. intonsa*, *F. occidentalis*, and *T. hawaiiensis*, adults were maintained and allowed to oviposit in custom frustum-shaped polypropylene cages (Cage A; top diameter 8 cm, bottom diameter 11 cm, height 8 cm). The cage top was covered with breathable mesh, and the bottom was sealed with Parafilm^®^ M (Bemis Company, Inc., Sheboygan Falls, WI, USA) and positioned above a water-filled tray to maintain membrane moisture while preventing direct water contact. Approximately 300 healthy adults (mixed sexes) were introduced into each cage (≥3 cages per species per batch), and germinated broad beans (*Vicia faba*) were supplied as food and replaced every 48 h to prevent microbial contamination. Females oviposited through the Parafilm into the membrane–water interface. Eggs were gently collected by rinsing the membrane three times with 10 mL of distilled water per rinse and subsequently vacuum-filtered using a SHZ-D(III) water-circulating vacuum pump (Zhengzhou Greatwall, Zhengzhou, China) onto black Whatman No. 1 filter paper (Cytiva, Marlborough, MA, USA) or black cotton cloth to enhance visibility. By contrast, *M. usitatus* showed poor adaptation to the Parafilm-based oviposition setup; therefore, germinated broad beans were placed directly inside the cage as the oviposition substrate. After 48 h, beans containing *M. usitatus* eggs and eggs of the other species collected from the Parafilm membranes were each transferred to their respective rectangular rearing cages (Cage B; 12 × 8 × 6 cm; fine-mesh sides) for hatching and nymphal development. A moistened filter paper sheet (Whatman No. 1, Cytiva, Marlborough, MA, USA) was placed on the cage bottom to maintain humidity and provide a pupation substrate, and both food and filter paper were replaced every 2 days. All thrips strains were maintained under the same controlled rearing conditions described above. Empty rearing cages and associated tools were sprayed with 70% ethanol and disinfected with UV light for 30 min before introducing the next generation. Developmental progress was monitored daily, and temperature and humidity were recorded using the built-in sensors of the environmental chamber to ensure stable rearing conditions. Individuals were collected at the designated developmental stages for subsequent analyses.

### 2.2. Morphometric Analysis and COI Reference Sequence Generation in Four Thrips Species

To ensure consistency across replicates and to eliminate variability caused by sexual dimorphism, ten adult females of each species were collected from laboratory-reared populations at 1–3 days post-eclosion. All individuals were fully sclerotized, morphologically intact, and free from visible deformities. Because pronounced sexual dimorphism in body size and external morphology exists between males and females, only females were examined for morphometric analysis to eliminate sex-related variability and ensure consistent and comparable measurements. This restriction was applied exclusively to the morphological measurements. Individual specimens were positioned at the center of a microscope slide for stereomicroscopic imaging. The wings were symmetrically aligned along the body axis in the same focal plane to minimize distortion caused by improper posture. Stereomicroscope imaging was performed using ZSA402 stereomicroscope (Chongqing Optec Instrument Co., Ltd., Chongqing, China). All images were calibrated using embedded 200 μm scale bars, and morphometric measurements were performed in millimeters (mm) using the S-EYE 2.0 image analysis system integrated with the stereomicroscope (Chongqing Optec Instrument Co., Ltd., Chongqing, China). All measurements were recorded to two decimal places.

All individuals used in this study were directly sampled from four well-established laboratory strains whose identity had been routinely verified through morphological examination and mitochondrial *COI* barcoding. At the start of the experimental workflow, species identity was further confirmed based on full-length mitochondrial *COI* sequence information prior to subsequent method development. For molecular characterization and generation of high-quality *COI* reference sequences, genomic DNA was extracted from 50 adult individuals randomly selected from laboratory-maintained strains, including both females and males, using a conventional buffer-based DNA extraction method (CB-Std), which consists of Tris–EDTA–NaCl buffer preparation, SDS-mediated lysis, and alcohol precipitation. Full-length mitochondrial *COI* fragments (approximately 1500 bp) from these individuals were then amplified and sequenced to generate high-quality templates for primer design. The extraction buffer consisted of 1.461 g NaCl (Solarbio, Beijing, China), 5 mL of 1 M Tris-HCl (Solarbio, Beijing, China), and 5 mL of 0.5 M EDTA (Solarbio, Beijing, China), which was adjusted to pH 8.0 and brought to a final volume of 50 mL with sterile ultrapure water (Milli-Q system; MilliporeSigma, Burlington, MA, USA). The buffer was stored at 4 °C. Each specimen was homogenized in a 1.5 mL microcentrifuge tube using a sterile micro-pestle. For lysis, 500 μL of extraction buffer and 100 μL of 10% SDS (Solarbio, Beijing, China) were added to each tube, followed by incubation at 56 °C for 1 h. Subsequently, 200 μL of 2 M potassium acetate (Solarbio, Beijing, China) was added, and the mixture was incubated at 4 °C for 1 h. After centrifugation at 14,000 rpm for 10 min, the supernatant was collected and mixed with an equal volume of pre-chilled isopropanol (Macklin Biochemical Co., Ltd., Shanghai, China; AR grade) to precipitate DNA. The resulting DNA pellet was air-dried, resuspended in 20 μL of sterile ultrapure water (Milli-Q system; MilliporeSigma, Burlington, MA, USA), and stored at −20 °C until use.

PCR amplification was carried out in a 25 μL reaction volume containing 12.5 μL of 2× Taq PCR Master Mix (Vazyme, Nanjing, China), 1 μL each of forward and reverse primers (10 μM), 1 μL of genomic DNA template, and nuclease-free water to a final volume of 25 μL. Thermal cycling conditions were as follows: initial denaturation at 95 °C for 3 min, followed by 35 cycles of 95 °C for 15 s, 58 °C for 15 s, and 72 °C for 30 s, with a final extension at 72 °C for 10 min. PCR products were verified by electrophoresis on a 2% agarose gel, and target fragments were excised and purified using a Gel Extraction Kit (Omega Bio-Tek, Norcross, GA, USA). Purified PCR fragments were ligated into the pJET1.2 vector using the CloneJET PCR Cloning Kit (Thermo Fisher Scientific, Waltham, MA, USA) and transformed into *Escherichia coli* DH5α competent cells (Tiangen Biotech Co., Ltd., Beijing, China). Transformed cells were plated on LB agar supplemented with ampicillin (100 μg/mL) and incubated at 37 °C for 12–16 h. Positive colonies were inoculated into LB broth containing ampicillin and incubated at 37 °C with shaking at 200 rpm for 6 h prior to plasmid extraction. Inserts were confirmed by PCR using pJET1.2 universal primers, and the products were validated by 1% agarose gel electrophoresis. Sequencing was carried out in both directions by Sangon Biotech (Shanghai, China). Raw sequence reads were assembled and manually curated using SnapGene (version 6.2.1; GSL Biotech LLC, San Diego, CA, USA; https://www.snapgene.com/), generating high-quality *COI* reference sequences for the four target thrips species. These verified sequences were subsequently used as templates for species-specific primer design and downstream analyses.

### 2.3. Sequence Alignment and Species-Specific Primer Design

Based on the obtained *COI* sequences, multiple sequence alignment and characterization were performed using MEGA software (version 12.0; Arizona State University, Tempe, AZ, USA). Given that *COI* is a protein-coding gene with a conserved reading frame, sequence alignment was primarily inspected and refined manually. Nucleotide composition (A%, T%, G%, and C%) and GC content were calculated to assess base composition bias and sequence complexity across the four thrips species. Pairwise genetic distances were estimated under the *p*-distance model, with missing data treated using pairwise deletion, resulting in a genetic distance matrix among species. To identify species-specific regions, the aligned *COI* sequences were further visualized and manually examined using SnapGene (version 6.2.1; GSL Biotech LLC, San Diego, CA, USA; https://www.snapgene.com/) to locate variable nucleotide sites. Regions exhibiting high polymorphism were annotated as potential targets for primer design.

During primer design, highly conserved regions were excluded to avoid reduced discriminatory power. Candidate primers were selected from the most variable *COI* segments, with priority given to those containing at least one species-specific nucleotide at the 3′ terminus. Design principles were as follows: (1) targeting regions with the highest nucleotide variability; (2) maintaining amplicon lengths between 600 and 800 bp; (3) avoiding regions with high homology to non-target species; and (4) minimizing the formation of hairpins, dimers, or self-complementarity. All primers were designed using Primer Premier (version 6.0; PREMIER Biosoft, Palo Alto, CA, USA; https://www.premierbiosoft.com/) and further checked in SnapGene (version 6.2.1; GSL Biotech LLC, San Diego, CA, USA; https://www.snapgene.com/) to confirm complete matching to the intended target region and to exclude predicted secondary structures in the primers. Final primer sequences are provided in Table S1.

### 2.4. Validation of Primer Specificity and Performance

Amplification efficiency and species specificity were evaluated by performing PCR assays using genomic DNA from the four thrips species under the same thermal cycling program as *COI* amplification. Amplicons were resolved on 1% agarose gels to verify the presence of the expected species-specific band. Each primer pair was tested against its cognate target and all non-target species. Primer pairs yielding a single, distinct band in the target species and no amplification in non-targets were considered specific and selected for further validation. Primer performance under mixed-template backgrounds was assessed using two mock DNA communities to simulate interspecific mixtures. The first mixture consisted of genomic DNA from *F. intonsa*, *F. occidentalis*, *M. usitatus*, and *T. hawaiiensis*, with each species contributing an equal amount to a total of 50 ng DNA per reaction. The second mixture contained only the three non-target species, with the total DNA amount likewise adjusted to 50 ng per reaction, excluding the target species for a given primer pair. Reactions were run under the same conditions as single species assays. Successful species discrimination was defined as amplification only in mixtures containing the target species, with no bands in target-absent mixtures.

### 2.5. Short-Turnaround DNA Extraction and Single Thrips Adult PCR Detection

To evaluate short-turnaround DNA extraction strategies suitable for single-insect diagnostics under resource-limited conditions, six extraction methods were systematically compared using individual adult thrips. These included a modified conventional protocol (CB-Std) and five short-turnaround alternatives designed to reduce procedural complexity while maintaining compatibility with downstream PCR.

The CB-Std method (Section 2.2) served as the reference protocol and involved SDS-based chemical lysis, potassium acetate precipitation, and DNA resuspension. For single-adult processing, the total reaction volume was reduced from 500 μL to 20 μL with proportional adjustment of reagents. The five short-turnaround extraction methods were classified according to buffer composition and lysis strategy. Three detergent-free methods employed sterile distilled water (SDW; Milli-Q system; MilliporeSigma, Burlington, MA, USA), phosphate-buffered saline (PBS; ABKBio, Xiamen Aibikang Biotechnology Co., Ltd., Xiamen, China), or 20 mM EDTA (Solarbio, Beijing, China) as lysis media. For these methods, individual thrips were homogenized in 20 μL of the respective buffer and subjected to brief heat lysis at 98 °C for 2 min, followed by cooling to room temperature. Importantly, the PBS-based protocol did not include SDS or any other detergents, and the resulting lysates were used directly as PCR templates without further purification. In contrast, a simplified detergent-assisted protocol (CB-SDS) utilized a reduced-concentration lysis buffer containing 0.2% SDS to evaluate the effect of mild detergent inclusion on DNA release and amplification efficiency. A nitrocellulose membrane contact transfer (NCM) method was also evaluated. In this approach, individual thrips were directly pressed onto nitrocellulose membranes (Solarbio, Beijing, China) and air-dried at room temperature. Prior to PCR, membrane fragments (approximately 0.5 × 0.5 cm) bearing the thrips contact trace were excised and transferred into 20 μL of sterile water (Milli-Q system; MilliporeSigma, Burlington, MA, USA). The thrips contact trace was defined as residual biological material transferred from the insect body surface during physical contact, including cuticular fragments, cellular debris, and trace amounts of internal tissue or hemolymph sufficient to serve as a DNA template. Samples were heated at 98 °C for 2 min to release DNA and then cooled to room temperature before PCR.

DNA concentration and purity were measured using a NanoDrop microvolume spectrophotometer (Thermo Fisher Scientific, Waltham, MA, USA). For each extraction method, ten biological replicates were tested per thrips species, resulting in a total of 240 PCR amplification reactions. Amplification performance was assessed by 1% agarose gel electrophoresis to compare band clarity, specificity, and PCR compatibility across extraction methods.

### 2.6. Determination of PCR Detection Sensitivity

Genomic DNA extracted from single adults of the four species was serially diluted to five concentration levels, corresponding to DNA inputs of 50 ng, 10 ng, 1 ng, 100 pg, and 10 pg per 25 μL PCR reaction. PCR amplification was conducted using the species-specific primer pairs designed for each thrips species, following the same reaction composition and thermal cycling parameters described in Section 2.2. The amplified products were resolved on 1% agarose gels, and band visibility and clarity were evaluated to assess amplification performance across DNA input levels. The lowest DNA amount consistently producing a visually distinct and reproducible band was defined as the limit of detection (LOD) for each assay. Each DNA input level was conducted in three biological replicates to confirm reproducibility and reliability.

### 2.7. Evaluation of Species-Specific Sensitivity Across Mixed Population Ratios

To assess the detection sensitivity of target thrips across varying abundance ratios, a gradient mixture assay was conducted. Gradient mixtures were prepared by combining one adult of the target species with one to four individuals of each of the other three species, yielding target-to-background ratios of 1:1:1:1, 1:2:2:2, 1:3:3:3, and 1:4:4:4. Corresponding negative controls were prepared using the same ratios but excluding the target species (non-target thrips only). Each mixture was subsequently homogenized in 50 μL of 1× PBS using the PBS-based short-turnaround extraction protocol, ensuring consistency among treatments. Each treatment was performed with three biological replicates, and 1 μL of extracted DNA was used per PCR reaction to maintain consistent template input across assays.

### 2.8. Statistical Analysis

Morphometric parameters (body length and width) and DNA extraction metrics (yield, purity, and storage stability) were expressed as mean ± standard deviation (SD) calculated from ten biological replicates (*n* = 10) per species. Statistical analyses were performed using IBM SPSS Statistics (version 26.0; IBM Corp., Armonk, NY, USA; https://www.ibm.com/products/spss-statistics (accessed on 14 January 2026)). One-way analysis of variance (ANOVA) was applied to compare means among treatments, followed by Tukey’s multiple comparison test, with significance set at *p* < 0.05. GraphPad Prism (version 9.0.0; GraphPad Software, San Diego, CA, USA; https://www.graphpad.com/) was used for data visualization and figure preparation.

## 3. Results

### 3.1. External Morphology and Interspecific COI Divergence Among Four Thrips Species

**Figure 1:**
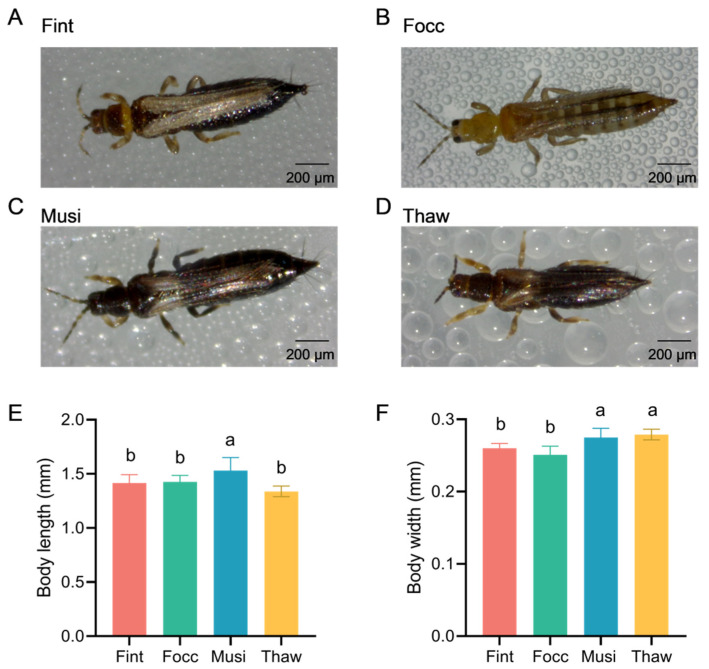
External morphology and body size comparison among four thrips species. (**A**–**D**) Representative adult females of Fint (*F. intonsa*); Focc (*F. occidentalis*); Musi (*M. usitatus*); Thaw (*T. hawaiiensis*) under stereomicroscopy. Scale bars: 200 μm. (**E**) Comparison of body length among the four species (*n* = 10 per species). (**F**) Comparison of body width among the four species (*n* = 10 per species). Bars sharing the same lowercase letter do not differ significantly, whereas bars labeled with different letters indicate statistically significant differences among species (one-way ANOVA followed by Tukey’s post hoc test, *p* < 0.05). Data are presented as mean ± standard deviation (SD).

Adult females of the four thrips species examined exhibit highly similar overall external morphology at the structural level under routine observation (Figure 1A–D), with no readily diagnostic structural characters distinguishable among species.

Simple body size measurements revealed limited but statistically significant differences in body length and width among species (Figure 1E,F). Specifically, *M. usitatus* exhibited a greater mean body length, whereas *M. usitatus* and *T. hawaiiensis* showed slightly increased body width compared with the other species (one-way ANOVA followed by Tukey’s post hoc test, *p* < 0.05). However, substantial overlap was observed across species for both parameters, indicating that these measurements alone provide insufficient resolution for reliable species-level identification. Although minor differences in pigmentation intensity were visually apparent among species, such traits are highly variable and strongly influenced by age, physiological condition, and environmental factors, and therefore cannot be considered reliable diagnostic characters under field conditions.

**Figure 2:**
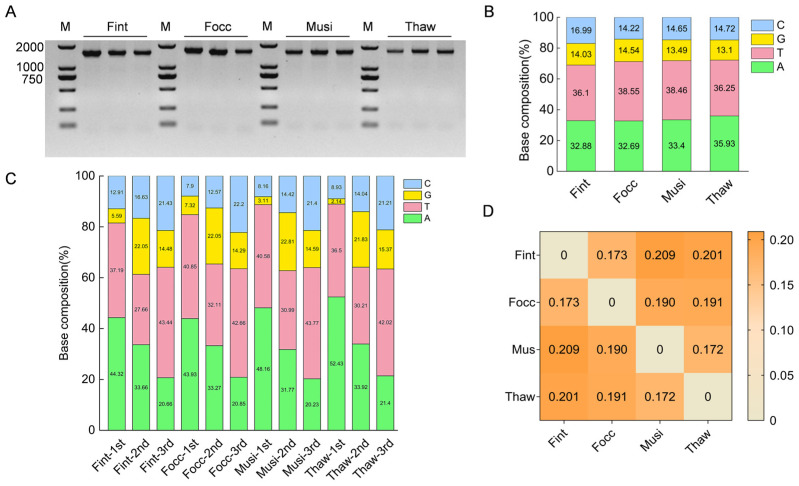
Sequence characterization of *COI* genes sequences in four thrips species. (**A**) Agarose gel electrophoresis showing successful amplification of full-length *COI* sequences in Fint (*F. intonsa*), Focc (*F. occidentalis*), Musi (*M. usitatus*), and Thaw (*T. hawaiiensis*). Representative amplicons of the expected size are shown for each species, confirming consistent *COI* amplification for subsequent sequencing analyses. M: DNA marker (DL2000). (**B**) Overall base composition of *COI* sequences, showing a strong AT bias in all four species. (**C**) Base composition by codon position, with pronounced AT enrichment at the first and third positions. (**D**) Pairwise genetic distances (uncorrected *p*-distance) among species, calculated based on full-length *COI* sequences.

To enable species-specific molecular identification, the mitochondrial *COI* gene was selected as the diagnostic target, and its interspecific variation among the four species was analyzed. PCR amplification of the *COI* gene produced bands migrating between the 1000 and 2000 bp markers and visually closer to the 2000 bp marker in all four thrips species (Figure 2A). Subsequent sequence analysis revealed that the amplicons were 1554 bp in *F. intonsa* and *F. occidentalis* and 1542 bp in *M. usitatus* and *T. hawaiiensis*, indicating minor interspecific length variation within the *COI* coding region that is not visually resolvable on agarose gels. Alignments to the corresponding NCBI references (*F. intonsa* NC_081965.1, *F. occidentalis* NC_018370.1, *M. usitatus* NC_070094.1, and *T. hawaiiensis* MW582621.1) showed high identity, with only a few nucleotide mismatches. Based on this verification, the experimentally derived *COI* sequences were used as templates for species-specific primer design and downstream analyses. Given the strong concordance with public entries, no additional GenBank submissions were made, and all downstream analyses were based on the experimentally derived *COI* sequences.

Base-composition analysis revealed a pronounced AT bias across all four species, with overall A + T content exceeding 68% (Figure 2B). Among the four species, *T. hawaiiensis* exhibited the highest adenine (A) content (35.93%), whereas *F. occidentalis* and *M. usitatus* showed the highest thymine (T) levels (38.55% and 38.46%, respectively). Guanine (G) was the least abundant nucleotide, accounting for only 13.10–14.54% of total bases. Interspecific differences were most evident for adenine (A) and cytosine (C), indicating species-specific nucleotide usage preferences.

Positional analysis further demonstrated that the first and third codon positions exhibited the strongest AT enrichment, with A + T content exceeding 80% (Figure 2C). At the first codon position, *T. hawaiiensis* showed A content as high as 52.43%, whereas G and C were markedly underrepresented. G was particularly scarce at this position, with minimum values of 5.59% in *F. intonsa* and 2.14% in *T. hawaiiensis*. The second codon position displayed a more balanced nucleotide distribution, consistent with stronger functional constraints on protein-coding regions. By contrast, the third codon position was strongly enriched in thymine, reaching 43.44% in *F. intonsa*, with the other species showing similar levels (approximately 42%). Collectively, these codon-specific patterns highlight diagnostic polymorphisms within the *COI* coding region that can serve as reproducible molecular markers for species discrimination.

Pairwise uncorrected *p*-distance analysis of the *COI* coding region revealed interspecific values ranging from 0.172 to 0.209 among the four thrips species (Figure 2D). The smallest *p*-distance (0.173) was observed between *F. intonsa* and *F. occidentalis*, whereas the largest value (0.209) occurred between *M. usitatus* and *T. hawaiiensis*, representing only a modest increase relative to the minimum. Overall, all interspecific comparisons showed *p*-distance values exceeding 0.17, indicating clear sequence divergence among species in the *COI* coding region. Because *p*-distance is an uncorrected metric, these values summarize observed sequence differences and may underestimate underlying evolutionary change if multiple substitutions have occurred. Taken together, these results demonstrate consistent interspecific *COI* sequence differences among all species pairs examined, providing a suitable basis for subsequent species identification and species-specific primer design in this study.

### 3.2. Species-Specific COI Primer Screening and Validation

To identify regions suitable for species-specific primer design, *COI* sequences from the four thrips species were aligned and analyzed for interspecific divergence patterns as described above (Figure S1). A polymorphism-rich region spanning positions 600–1300 bp, marked by dense nucleotide substitutions and pronounced interspecific variation, was identified and selected as the target region for primer development. Within this region, three candidate primer pairs were designed for each of the four thrips species in accordance with standard primer design principles, resulting in twelve primer sets in total (Table S1).

**Figure 3:**
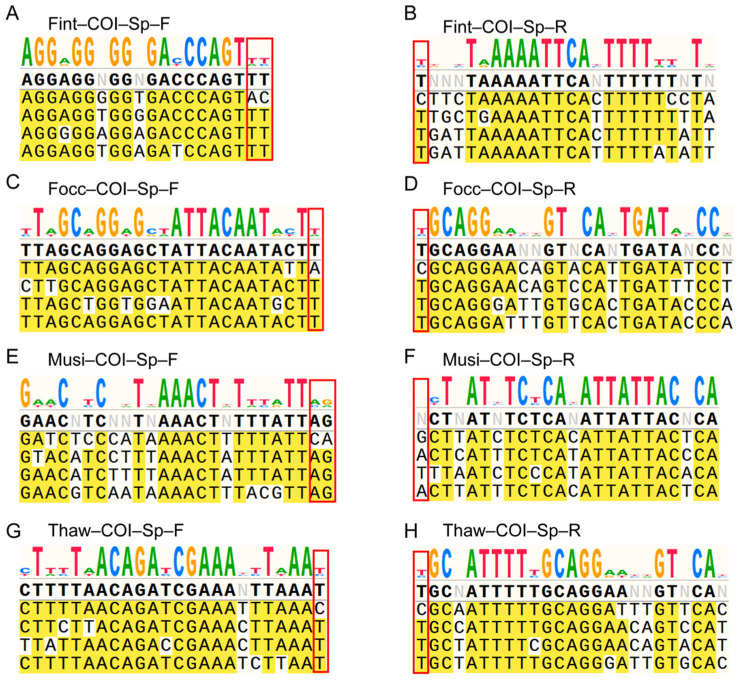
Primer binding regions and interspecific *COI* variation among four thrips species. (**A**–**H**) The upper panel shows a sequence logo summarizing nucleotide variability across aligned *COI* sequences from the four thrips species within the displayed window. The lower panel shows a multiple sequence alignment of representative *COI* sequences from the four species; the first sequence in each alignment corresponds to the species-specific primer designed for the target species indicated in the panel title, followed by the *COI* sequences of the four thrips species. Red boxes highlight the 3′-terminal region of the primer-binding site that was emphasized during primer design to ensure species specificity. The color scheme and bold formatting are automatically generated by the visualization software and do not indicate additional biological annotations.

Pooled genomic DNA extracted from 50 adult individuals per species was used as the template for primer screening to ensure experimental consistency and reproducibility. All candidate primers were first tested under identical PCR conditions using single species DNA templates to assess amplification performance and specificity, and primer pairs yielding weak or non-specific bands were excluded from further analyses (Figure S2). Subsequently, mixed-template assays containing DNA from multiple species were conducted to further evaluate potential cross-reactivity and confirm primer specificity (Figure S3). Only primer pairs that produced a single distinct amplicon in the presence of their respective target DNA, without any cross-reactive bands in non-target mixtures, were retained. After two rounds of screening under both single and mixed template conditions, one optimal primer pair was identified for each species and was subsequently renamed based on its species designation. The selected primer pairs annealed to *COI* coding regions exhibiting the greatest interspecific nucleotide divergence and met all predefined design criteria. Notably, within each primer pair, both the forward and reverse primers carried at least one diagnostic nucleotide at the 3′ terminus uniquely matching the target species, thereby ensuring high specificity and robust discriminatory power (Figure 3).

**Figure 4:**
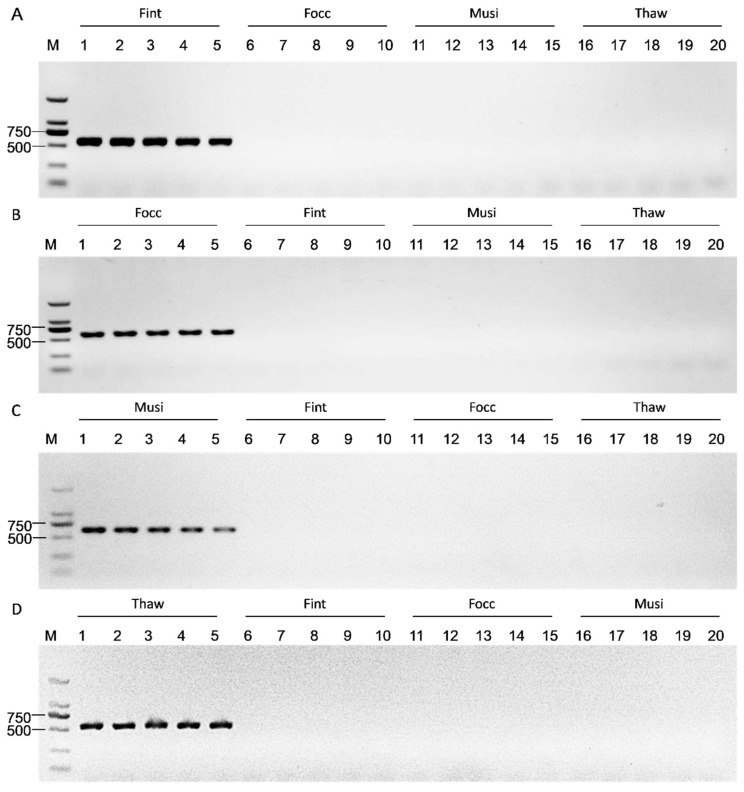
Species-specific PCR amplification with *COI* primers under single species template conditions. (**A**) Fint (*F. intonsa*); (**B**) Focc (*F. occidentalis*); (**C**) Musi (*M. usitatus*); (**D**) Thaw (*T. hawaiiensis*). A single, distinct amplicon was observed exclusively in its corresponding species, with sizes of 580 bp for Fint, 590 bp for Focc, 652 bp for Musi, and 561 bp for Thaw. No cross-reactivity was observed. Qualitative validation of primer specificity based on amplification presence or absence rather than visual comparison of interspecific differences. All PCR assays were independently repeated five times with consistent results. M: DNA marker (DL2000).

Each optimized primer pair was further validated and produced a single, distinct amplicon of the expected size in its corresponding target species (580 bp in *F. intonsa*, 590 bp in *F. occidentalis*, 652 bp in *M. usitatus*, and 561 bp in *T. hawaiiensis*), whereas no amplification was detected in the non-target species (Figure 4).

**Figure 5:**
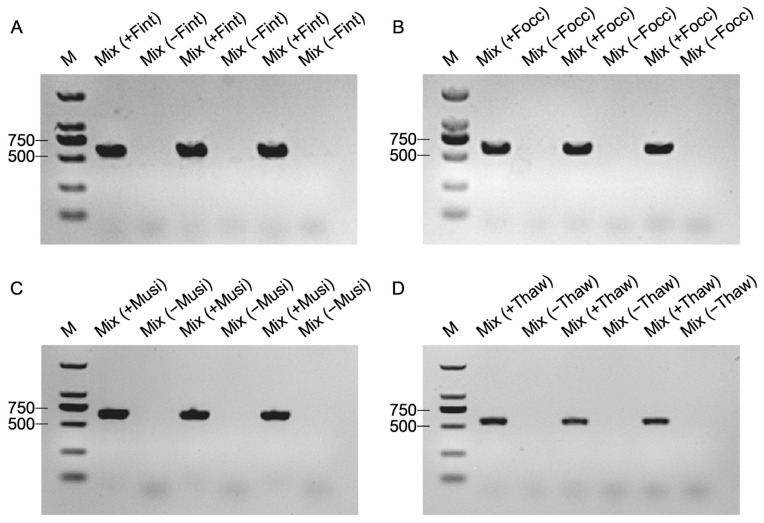
Species-specific PCR amplification using a mixture of single species DNA templates. (**A**) Fint (*F. intonsa*), 580 bp; (**B**) Focc (*F. occidentalis*), 590 bp; (**C**) Musi (*M. usitatus*), 652 bp; (**D**) Thaw (*T. hawaiiensis*), 561 bp. For each panel, three independent biological replicates are shown for both “+” and “−” mixtures. PCR assays were performed using species-specific primers with mixed DNA templates (total 50 ng DNA per reaction). “+”: target species included; “–”: excluded. Distinct amplicons were detected exclusively in “+” mixtures, with no cross-amplification observed in “–” groups. M: DNA marker (DL2000).

Subsequently, PCR assays using mixed DNA templates (a total of 50 ng DNA per reaction, with equal contributions from each species), either containing or lacking the target species, yielded clear single bands exclusively in mixtures containing the respective targets (Figure 5). These results confirm that the optimized *COI* primer system enables species-specific and reproducible discrimination of closely related thrips species under mixed-species backgrounds.

### 3.3. Optimization and Performance Assessment of DNA Extraction Methods for Single Thrips Adult Detection

Based on the establishment of species-specific *COI* primers capable of accurately distinguishing four thrips species, six DNA extraction protocols were systematically evaluated for single insect samples to enhance their field-level diagnostic utility. Accordingly, five non-instrumental lysis methods (PBS, EDTA, SDW, NCM, and CB-SDS) were evaluated, with CB-Std employed as the positive control.

**Figure 6:**
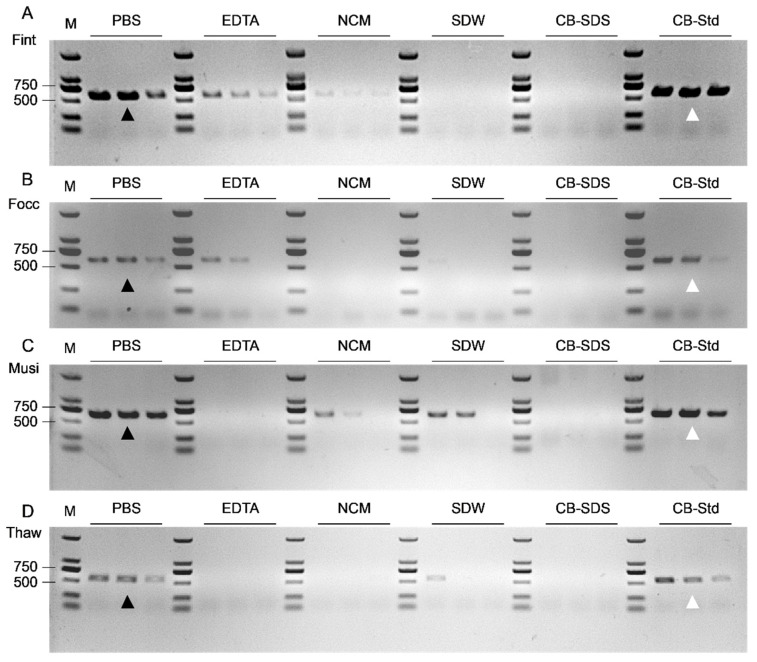
PCR performance of six DNA extraction methods for single thrips adult. (**A**) Fint (*F. intonsa*, 580 bp). (**B**) Focc (*F. occidentalis*, 590 bp). (**C**) Musi (*M. usitatus*, 652 bp). (**D**) Thaw (*T. hawaiiensis*, 561 bp). Week 0 PCR products of single thrips adult extracted using PBS, EDTA, NCM, SDW, CB-SDS, and CB-Std are shown. Black triangles indicate PBS as the optimal short-turnaround method, and white triangles indicate CB-Std as the standard control. M: DNA marker (DL2000).

Representative amplification patterns, classified as strong, moderate, and weak, are presented (Figure 6). Immediately after DNA extraction (week 0), the PBS and CB-Std groups produced distinct species-specific bands (indicated by arrowheads) across all four thrips species, yielding clear and reproducible amplicons of the expected size, with a 100% amplification success rate (40/40; Figure 6 and Figure S4). In contrast, the remaining four rapid methods exhibited markedly lower efficiency, with success rates generally below 25% and poor amplification clarity and reproducibility (Figure 6, Figure S5 and S6). Accordingly, these four methods were deemed unsuitable for downstream PCR applications, both after 1–4 weeks of refrigerated storage and for molecular detection of single thrips adult. Subsequent weekly PCR validations revealed that both PBS and CB-Std maintained clear and consistent amplification during the first two weeks. However, from week 3 onward, band intensities for *F. occidentalis* and *T. hawaiiensis* gradually weaken under both extraction methods, with one or two samples failing to produce detectable amplicons. By week 4, this trend became more pronounced, particularly in *T. hawaiiensis*, where bands became increasingly faint, although the overall amplification success rate still remained above 80% (Figures S7–S10).

**Figure 7:**
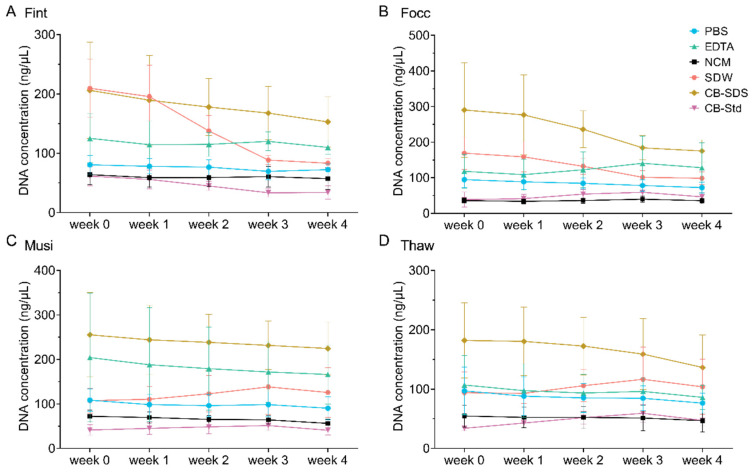
DNA concentration dynamics of four thrips species under refrigerated storage based on six extraction methods. (**A**) Fint (*F. intonsa*); (**B**) Focc (*F. occidentalis*); (**C**) Musi (*M. usitatus*); (**D**) Thaw (*T. hawaiiensis*). DNA concentrations (ng/μL) were measured weekly over a four-week period with six extraction buffers: PBS, EDTA, NCM, SDW, CB-SDS, and CB-Std. Data represent mean ± standard deviation (SD) from ten biological replicates.

To assess DNA preservation under refrigerated conditions, DNA concentration and purity were monitored from week 0 to week 4 (Figure 7). At week 0, CB-SDS yielded the highest DNA concentrations, ranging from 194.68 ± 38.52 ng/μL (*T. hawaiiensis*) to 290.41 ± 133.13 ng/μL (*F. occidentalis*). EDTA yielded concentrations between 111.52 ± 44.85 ng/μL (*T. hawaiiensis*) and 204.11 ± 144.56 ng/μL (*M. usitatus*), whereas SDW ranged from 87.84 ± 26.17 ng/μL (*T. hawaiiensis*) to 209.69 ± 48.64 ng/μL (*F. intonsa*), both representing intermediate levels. PBS produced moderate but highly reproducible concentrations (80.61–108.42 ng/μL, with SDs mostly below 25 ng/μL except in *T. hawaiiensis*), indicating good consistency across species. NCM yielded DNA concentrations ranging from 35.81 ± 7.37 ng/μL in *F. occidentalis* to 72.15 ± 14.05 ng/μL in *M. usitatus*, while CB-Std ranged from 34.00 ± 4.66 ng/μL in *T. hawaiiensis* to 61.50 ± 16.11 ng/μL in *F. intonsa*. During the four-week storage period, DNA concentrations obtained using PBS remained above 69.60 ng/μL. By contrast, CB-Std consistently yielded low concentrations (approximately 33.46–61.50 ng/μL) with minimal variation, indicating high reproducibility despite limited DNA recovery. CB-SDS, however, exhibited a marked decline in DNA concentration starting at week 1, accompanied by increased variability among samples. EDTA and SDW showed a more gradual downward trend over the same period, while NCM consistently produced the lowest concentrations, indicating limited potential for long-term storage.

Analysis of A260/A280 and A260/A230 ratios revealed method-specific temporal trends in DNA absorbance profiles over time (Figures S11 and S12). At week 0, CB-Std showed the highest A260/A280 ratios (ranging from 2.22 ± 0.07 in *T. hawaiiensis* to 2.49 ± 0.20 in *F. occidentalis*) but consistently low A260/A230 values (ranging 0.29 ± 0.33 in *F. intonsa* to 0.60 ± 0.30 in *T. hawaiiensis*), suggesting RNA carryover and residual organic or chaotropic contaminants. In contrast, PBS exhibited the most balanced absorbance profile, with A260/A280 ratios ranging from 1.41 ± 0.11 (*F. occidentalis*) to 1.58 ± 0.14 (*M. usitatus*) and A260/A230 ratios ranging from 0.59 ± 0.05 (*F. intonsa*) to 0.67 ± 0.10 (*F. occidentalis*), reflecting relatively stable UV absorbance characteristics of the crude extracts. Throughout the storage period, CB-Std maintained high A260/A280 ratios (approximately 2.2–2.5); however, A260/A230 ratios showed a progressive decline in most species, dropping below 0.20 by week 4 in *F. occidentalis*, *M. usitatus*, and *T. hawaiiensis*, suggesting co-extraction of residual impurities and potential DNA degradation. By contrast, PBS maintained both ratios within relatively narrow ranges, with A260/A280 values between 1.39 and 1.64 and A260/A230 values between 0.54 and 0.73, showing minimal week-to-week variation (SD < 0.15) and demonstrating superior stability of absorbance profiles over the storage period. NCM displayed a similar yet slightly lower profile, with moderate and stable values across both ratios. EDTA consistently produced moderately low A260/A280 ratios (approximately 1.18–1.62) and uniformly low A260/A230 ratios (below 0.32) across all species, consistent with interference from residual chelating agents. SDW and CB-SDS yielded the lowest and most variable values for both ratios, particularly after week 2, suggesting compromised DNA quality and possible accumulation of inhibitory substances. Taken together, PBS exhibited the highest stability and reproducibility in DNA absorbance ratios and DNA yield across extraction methods and species, highlighting its utility as a reproducible and field-compatible buffer for single insect molecular identification.

### 3.4. Validation of Detection Sensitivity for the Species-Specific PCR System

**Figure 8:**
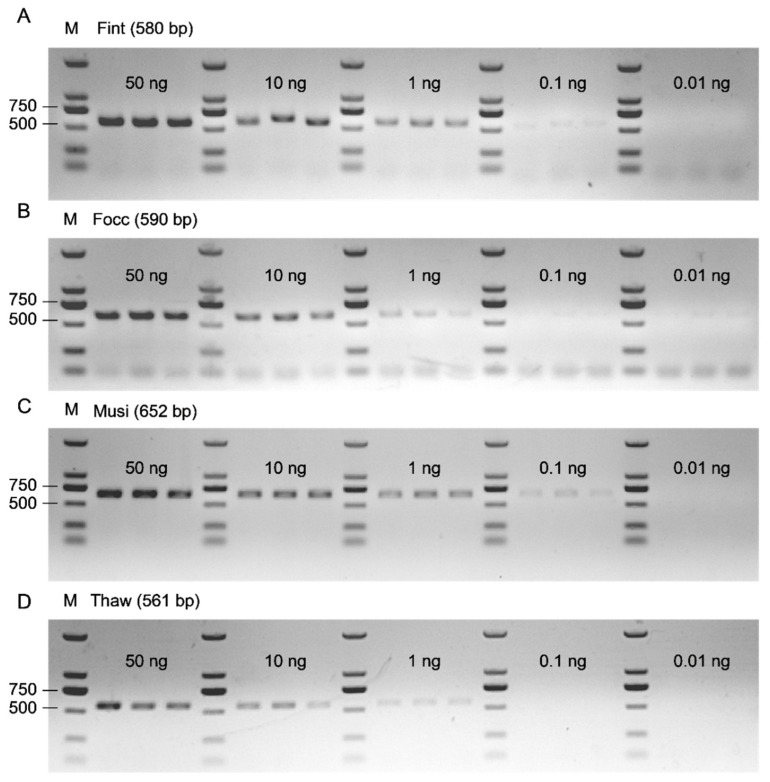
Detection sensitivity of the species-specific PCR assay in four thrips species. (**A**–**D**) Amplification profiles of Fint (*F. intonsa*, 580 bp), Focc (*F. occidentalis*, 590 bp), Musi (*M. usitatus*, 652 bp), and Thaw (*T. hawaiiensis*, 561 bp). PCR was performed using PBS-extracted genomic DNA at five DNA input levels (50, 10, 1, 0.1, and 0.01 ng per 25 μL; 1 μL input). Clear, single, species-specific bands were consistently observed at ≥1 ng, with faint or sporadic bands at 0.1 ng in Fint and Musi, and no detectable bands at 0.01 ng. M, DNA marker (DL2000).

To determine the detection sensitivity of the PCR assay, PBS-extracted genomic DNA was tested at five DNA input levels (50, 10, 1, 0.1, and 0.01 ng per 25 μL reaction) across four thrips species, with three biological replicates per species. All primer pairs consistently yielded single, species-specific amplicons of the expected sizes at DNA inputs ≥ 1 ng, with no off-target amplification observed (Figure 8A–D). Collectively, these results define 1 ng per reaction as the LOD for specific and reproducible amplification. The sporadic amplification at 0.1 ng in *F. intonsa* and *M. usitatus* delineates a practical detection window of 0.1–1 ng for single thrips adult. Minor interspecific differences were observed at low template levels, reflecting slight variation in amplification efficiency. Importantly, these differences did not compromise the overall diagnostic performance of the assay.

### 3.5. Effect of Species Background and Sample Input on PCR Inhibition and Its Alleviation by Dilution

**Figure 9:**
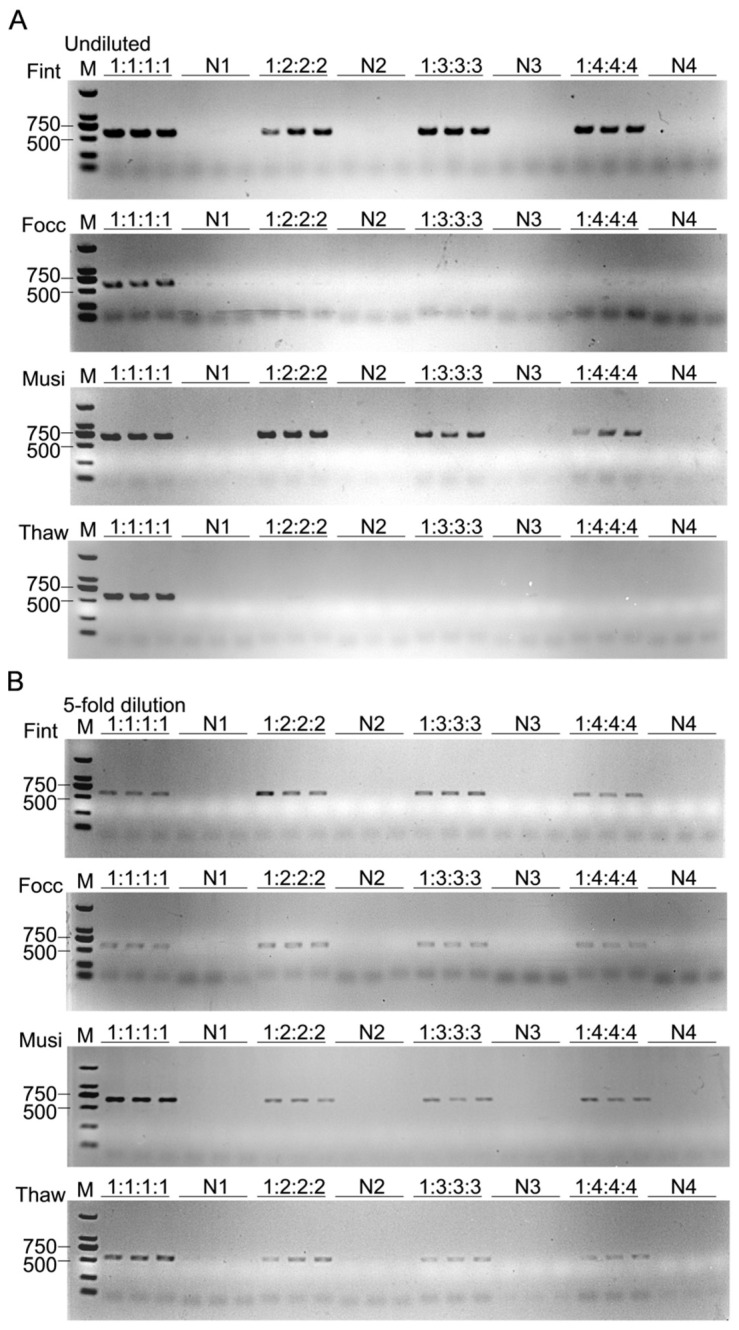
Species-specific amplification of the PCR assay under mixed-species background interference. (**A**) Undiluted lysates. (**B**) Fivefold-diluted lysates. Each lane contains PBS-extracted genomic DNA from a mixture of one individual of the target species (*F. intonsa*, *F. occidentalis*, *M. usitatus*, or *T. hawaiiensis*) and one to four individuals of each of the three non-target species, forming target-to-background ratios of 1:1:1:1, 1:2:2:2, 1:3:3:3, and 1:4:4:4. M: DNA marker (DL2000); N1–N4, mixtures containing only non-target species at the same ratios.

To evaluate species-specific amplification under mixed-species backgrounds, one adult of the target thrips was combined with one to four individuals of each of the three non-target species, forming target-to-background ratios of 1:1:1:1, 1:2:2:2, 1:3:3:3, and 1:4:4:4. Genomic DNA from each mixture was extracted with PBS. In undiluted reactions, *F. intonsa* and *M. usitatus* consistently yielded distinct, species-specific amplicons across all ratios. By contrast, *F. occidentalis* and *T. hawaiiensis* were detectable only at the ratio of 1:1:1:1, and their signals progressively attenuated and eventually disappeared as the proportion of non-target individuals increased. Following fivefold dilution, specific amplification was restored for all four species irrespective of mixing ratio (Figure 9). This recovery suggests that PCR inhibition, probably resulting from impurities or template competition in crude lysates, was effectively mitigated by dilution. Consistent results from three biological replicates indicate that the assay is reproducible and suitable for detecting targets at low abundance within complex and field-relevant backgrounds.

**Figure 10:**
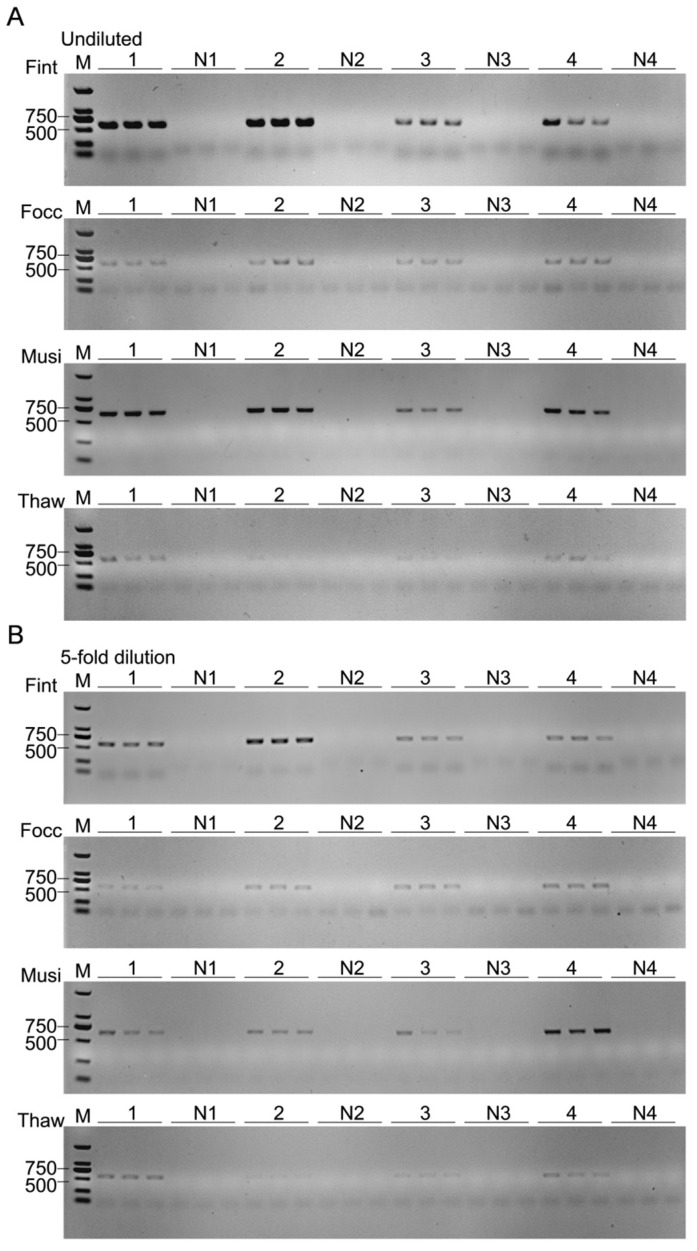
Interspecific variation in PCR inhibition. (**A**) Undiluted lysates. (**B**) Fivefold-diluted lysates. Samples were prepared by pooling 1–4 individuals of *Fint* (*F. intonsa*), *Focc* (*F. occidentalis*), *Musi* (*M. usitatus*), or *Thaw* (*T. hawaiiensis*). PBS-extracted crude DNA was used as the template. 1–4, number of pooled individuals; N1–N4, no-template negative controls corresponding to each gradient. M, DNA marker (DL2000).

To assess interspecific differences in lysate-induced effects on amplification efficiency, single species abundance-gradient assays were conducted using PBS-prepared crude lysates. Crude lysates from *F. intonsa*, *F. occidentalis*, and *M. usitatus* consistently produced clear amplicons across samples containing one to four individuals, and these results remained unchanged after fivefold dilution, indicating relatively weak inhibitory effects. In contrast, *T. hawaiiensis* exhibited a strong, non-linear inhibition pattern, with markedly reduced amplification in two-individual samples, where bands became nearly undetectable. Signals were slightly recovered at higher inputs, suggesting that inhibition intensity varied with sample composition. After uniform fivefold dilution, the overall amplification pattern remained similar, indicating that dilution had limited effects on relieving inhibition in *T. hawaiiensis* lysates (Figure 10). These findings highlight pronounced interspecific variation in lysate-induced inhibition, with *T. hawaiiensis* showing the strongest and most persistent suppression among the four species, consistent with a higher abundance of PCR inhibitors in its crude lysates.

## 4. Discussion

The high taxonomic diversity and pronounced morphological similarity of thrips communities limit the reliability of conventional morphology-based identification, particularly when specimens are fragile or collected at variable developmental stages [27,28,29]. To address these practical limitations, we established a species-specific molecular identification framework targeting the mitochondrial *COI* region, which enables reproducible, target-specific amplification in both individual and mixed-species samples. Unlike conventional DNA barcoding approaches that rely on universal primers targeting conserved regions, followed by post-PCR sequencing and subsequent sequence alignment [30,31], our system employs a polymorphism-informed, 3′-terminal discriminatory primer design anchored in species-specific polymorphic sites, enabling selective amplification in combination with a PBS-based short-turnaround DNA extraction protocol that minimizes procedural complexity and enhances field compatibility. The resulting framework integrates a balance among analytical specificity, operational simplicity, and dilution-mitigated inhibition, providing a species-specific and reproducible molecular identification framework that is time-efficient compared with sequencing-based barcoding for thrips species of agricultural importance.

The mitochondrial *COI* gene, characterized by a combination of conserved and hypervariable regions, remains the most widely used molecular marker for animal species identification owing to its robust interspecific resolution [32,33]. In this study, alignment of *COI* sequences revealed polymorphism-enriched regions that supported species-specific discrimination while maintaining amplification efficiency. Although conventional DNA barcoding approaches based on universal primers and sequence alignment have greatly facilitated reference library development, they frequently suffer from cross-species amplification and ambiguous results in complex, mixed-species samples [34,35]. To overcome these limitations, we employed a polymorphism-guided, primer-level engineering strategy by introducing diagnostic substitutions at the 3′ terminus, leveraging the high sensitivity of DNA polymerase to terminal mismatches. Previous studies have demonstrated that even a single 3′-terminal mismatch can markedly inhibit polymerase extension, thereby preventing non-target amplification [36,37]. Accordingly, our terminal-mutation-driven primers yielded single, distinct amplicons for all four thrips species, with no detectable nonspecific products under mixed-template conditions. This design achieves a dynamic balance between specificity and sensitivity and demonstrates excellent reproducibility across biological replicates. Given that terminal mismatch discrimination is a universal property of DNA polymerase, this polymorphism-guided strategy has broad applicability across taxonomic groups. Because the diagnostic target is mitochondrial *COI*, which is present in both females and males, the molecular assay itself is not inherently sex-specific and is therefore applicable to both sexes in molecular identification contexts. Beyond thrips, this approach provides a scalable framework for the short-turnaround identification of other small-bodied, morphologically similar pest species and represents a conceptual shift from conserved-region-dependent barcoding to polymorphism-guided molecular diagnosis [38].

Sample preparation remains a primary bottleneck limiting the field-compatible deployment of molecular species identification workflows [39,40]. Although conventional CTAB protocols and commercial kits yield high-purity DNA, they are labor-intensive, reagent-intensive, and require specialized equipment, rendering them unsuitable for time-efficient, field-oriented workflows [41]. In contrast, the PBS-based crude extraction method demonstrated the highest success rate in direct PCR, likely attributable to its isotonic nature, buffering capacity, and high compatibility with downstream molecular reactions. This PBS-based protocol simplifies sample preparation by eliminating multi-step DNA purification and centrifugation, thereby improving compatibility with direct PCR from single insects. Standard PBS is an isotonic buffer containing Na^+^, K^+^, and phosphate but lacking divalent cations, providing a chemically stable environment during crude lysis. PBS can dilute potential PCR inhibitors and stabilize pH, while high-temperature incubation (98 °C) simultaneously facilitates efficient nucleic acid release and denatures endogenous proteins, including nucleases, thereby reducing nucleic acid degradation and improving amplification reproducibility [42,43,44]. These combined chemical and thermal effects synergistically enhance the compatibility of PBS lysates with standard DNA polymerase, enabling stable and specific amplification without purification [45]. Collectively, these mechanisms reduce reliance on conventional purification procedures, streamline sample processing, and minimize nucleic acid loss during handling [46]. Thus, PBS effectively preserves nucleic acid integrity while simplifying sample handling and enhancing assay compatibility, thereby providing reproducible templates for downstream amplification even under unpurified conditions.

Sensitivity is a key performance metric for the molecular species identification framework and is commonly quantified by the LOD, defined as the lowest target concentration that can be detected with acceptable reliability [47]. In this study, clear and reproducible amplicons were consistently obtained at a template input of 1 ng, and faint but detectable bands were observed in some thrips samples at 0.1 ng, suggesting that the LOD falls within the theoretical sensitivity range of conventional PCR, where 1–1000 ng of DNA template is typically considered workable [48]. Despite potential carryover of inhibitors from the short-turnaround lysis procedure, single, distinct amplicons were consistently obtained across all four thrips species, demonstrating that PCR inhibition was effectively alleviated under the tested dilution conditions. Although amplification remained reproducible overall, slight interspecific differences in band intensity were observed, suggesting that amplification efficiency may also be influenced by intrinsic sequence composition and structural features. GC-rich regions tend to form stable secondary structures, such as hairpins and G-quadruplexes, which hinder DNA polymerase extension under low-template conditions and thereby introduce amplification bias [49]. At limiting template levels, amplification efficiency becomes highly sensitive to primer–template binding kinetics, and even minor mismatches near the 3′ terminus can impair polymerase initiation, further amplifying interspecific variation in amplicon yield. Collectively, these molecular mechanisms plausibly account for the species-specific variation observed in amplification and indicate that the system achieves a favorable balance between sensitivity and stability.

In complex field-derived samples, the coexistence of non-target individuals and interspecific differences in metabolic profiles may introduce diverse PCR inhibitors that interfere with amplification efficiency [24,50]. To mimic this complexity, gradient mixtures of multiple thrips species were constructed to systematically evaluate the alleviation of PCR inhibition under mixed-template scenarios. Pronounced matrix-associated inhibition was observed but proved reversible. Under undiluted conditions, stable amplification was consistently observed for *F. intonsa* and *M. usitatus*, whereas *F. occidentalis* and *T. hawaiiensis* exhibited progressively weaker or undetectable bands as the proportion of non-target species increased. Following fivefold dilution, clear and distinct species-specific bands were restored for all four species, indicating that the inhibitory effect primarily resulted from excessive inhibitor concentrations in the crude lysate and confirming the pivotal role of dilution in alleviating PCR inhibition [51]. In thrips sample matrices, PCR inhibition likely arises from multiple sources acting in combination, broadly encompassing insect-derived and host plant-derived factors [24,25]. Insect-derived inhibitors include melanin and its precursors such as polyphenol oxidation products, chitin fragments, and protein residues, which can form nonspecific interactions with DNA polymerase and Mg^2+^ ions, thereby reducing enzymatic activity [24,52,53]. Additionally, components of the hemolymph phenoloxidase cascade may persist after lysis and suppress polymerase reactions [54]. This hypothesis was further supported by the single species gradient experiment, which confirmed that lysates from *F. intonsa*, *F. occidentalis*, and *M. usitatus* exerted minimal inhibitory effects on PCR, whereas *T. hawaiiensis* exhibited a non-linear amplification trend, characterized by the weakest signal at intermediate sample sizes and partial recovery at higher inputs. This pattern likely arises from the opposing effects of increasing template concentration and the accumulation of inhibitory substances in crude lysates, leading to a concentration-dependent equilibrium between amplification and suppression. After moderate dilution, the same trend persisted with overall weaker intensity, indicating that both template and inhibitor concentrations jointly determine the amplification outcome. These observations suggest that PCR inhibition in thrips lysates is influenced not only by template complexity but also by differences in biochemical composition among species, which may modulate the balance between amplification efficiency and inhibitory effects.

The alleviation of PCR inhibition observed under PBS-based extraction and moderate dilution conditions is consistent with well-established principles of PCR chemistry. Compared with lysis buffers containing chelating agents or surfactants, PBS introduces fewer exogenous compounds that may interfere with polymerase activity, allowing amplification outcomes to more directly reflect endogenous sample components [25,42]. It is well recognized that PCR inhibitors can reduce polymerase efficiency through nonspecific interactions with the enzyme or essential reaction ions such as Mg^2+^ [24,55]. Dilution reduces the effective concentration of such inhibitors and has been widely applied as a practical means to improve amplification performance in crude extracts [52,56,57]. In this context, the improved amplification observed here is in line with existing knowledge that a balance between template availability and inhibitor load determines PCR success. Together, these observations illustrate the practical utility of combining PBS-based extraction with dilution for enhancing amplification compatibility in complex insect-derived samples.

Beyond these methodological advances in DNA extraction and inhibitor management, this system has substantial practical relevance for pest thrips management. In the context of routine surveillance and large-scale monitoring, species-resolved detection supports early recognition of invasive species, real-time surveillance of population dynamics, and timely adjustment of biological control strategies and insecticide applications [58,59,60]. This capability is important because thrips species differ in their patterns of reproductive advantage and competitive displacement, making accurate identification before visible field symptoms appear critical for minimizing crop losses and avoiding unnecessary chemical inputs. Although adult thrips can often be identified morphologically by trained taxonomists under laboratory conditions, accurate determination becomes challenging during early infestations, in mixed-species samples, or in large-scale monitoring associated with plant trade and seedling circulation chains. Under such circumstances, the compatibility of the assay with single-adult crude lysates enables standardized and reproducible species-level identification without slide preparation or reliance on specialist taxonomic expertise, thereby facilitating routine monitoring during seedling circulation, flower transport, and trans-regional crop movement, which represent major dissemination pathways for *F. occidentalis* and other invasive thrips species and directly linking species-level diagnosis to management decisions.

Despite being reproducible under mixed-species backgrounds and strong inhibitor tolerance demonstrated across both laboratory- and field-derived samples, certain overarching limitations remain. One limitation of the present study lies in the reliance of primer design on *COI* haplotypes representing dominant regional populations, a constraint that ultimately reflects the incomplete geographic coverage and taxonomic representation of publicly available *COI* reference databases. Although additional haplotypes from geographically distant regions have been reported, comprehensive incorporation of all global variants was beyond the scope of this work. This limitation is not unique to the present study but is inherent to molecular species identification frameworks based on mitochondrial *COI* markers, particularly those employing species-specific primer sets. When uncharacterized haplotypes or closely related taxa are encountered, such assays may exhibit non-amplification or, in rare cases, erroneous assignment, underscoring the importance of continued expansion and curation of reference databases. Nevertheless, within the populations examined here, the molecular species identification framework demonstrated species-specific and reproducible amplification across both single-species and mixed-species samples, supporting its practical applicability to laboratory-based surveillance workflows. Future expansion of geographic sampling and haplotype diversity will further extend the applicability of this molecular species identification framework beyond the four thrips species validated in this study [61,62,63]. Moreover, the assay still relies on thermocycler-based PCR and has yet to achieve true portability or real-time field-oriented workflow. Although the PBS-based protocol simplifies sample preparation, it still requires brief thermal treatment, which currently limits its full deployment under equipment-free field conditions. To overcome these constraints, emerging visual isothermal amplification technologies, such as RPA-Cas12a, have shown great promise for field-compatible molecular identification, combining minimal instrument requirements with high analytical sensitivity demonstrated across diverse insect pest and pathogen systems [64,65,66]. Integrating this detection framework with portable readout devices could enable a field-compatible molecular platform that automates workflows from molecular identification to pest surveillance, thereby advancing molecular identification tools toward field-adaptable and data-driven applications.

## 5. Conclusions

In summary, we developed a *COI*-based molecular identification framework that provides species-specific amplification with reproducible performance under mixed-species backgrounds, while reducing operational complexity and minimizing dependence on column-based DNA purification. This polymorphism-guided framework can be scalable to other minute pests and further adapted to visual isothermal amplification or CRISPR-based platforms, thereby providing a field-compatible basis for developing instrument-free, field-adaptable molecular diagnostics that support earlier and more targeted pest management decisions.

## Data Availability

The data presented in this study are available upon reasonable request from the corresponding author.

## Supplementary Materials

The following supporting information can be downloaded at: https://www.mdpi.com/article/10.3390/biology15020171/s1, Figure S1: Sequence alignment of *COI* gene fragments from four thrips species; Figure S2: Primer screening using single-species DNA templates; Figure S3: Validation of primer specificity under mixed-template conditions; Figure S4: PCR amplification performance of PBS and CB-Std extraction methods at week 0 across four thrips species; Figure S5: PCR amplification performance of NCM and EDTA extraction methods at week 0 across four thrips species; Figure S6: PCR amplification performance of SDW and CB-SDS extraction methods at week 0 across four thrips species; Figure S7: PCR amplification performance of PBS and CB-Std extraction methods at week 1 across four thrips species; Figure S8: PCR amplification performance of PBS and CB-Std extraction methods at week 2 across four thrips species; Figure S9: PCR amplification performance of PBS and CB-Std extraction methods at week 3 across four thrips species; Figure S10: PCR amplification performance of PBS and CB-Std extraction methods at week 4 across four thrips species; Figure S11: Temporal variation of A260/A280 absorbance ratios across extraction methods; Figure S12: Temporal variation of A260/A230 absorbance ratios across extraction methods; Table S1: List of primers employed in molecular characterization.
