# Supplementary material for: A Species-Specific COI PCR Approach for Discriminating Co-Occurring Thrips Species Using Crude DNA Extracts
Source: Biology (Basel). 2026 Jan 17;15(2):171. doi: 10.3390/biology15020171 (PMC12838139; doi:10.3390/biology15020171)
Supplement: Supplementary file 1 [file biology-15-00171-s001.zip › biology-4034973-supplementary.pdf]

Table S1. List of primers employed in molecular characterization

Primer	Sequence (5′-3′)	Description
Fint–COI–F	ATGAATAAACATTTATATATTCTTA	Primers designed for full-length COI amplification across four thrips species
Fint–COI–R	TTAAAAGTTATAAATTTTTGGATT	
Focc–COI–F	ATGTATAAAAACAATTTTTTAATAA	
Focc–COI–R	TTAAAAGTTAAAAATCTTTGGATT	
Musi–COI–F	ATGTATTTTACGAAATGACTTTTTTC	
Musi–COI–R	TTATCTTTTTAAAACTTTAAAATT	
Thaw–COI–F	ATAACAAAATTAGAGAAATGACT	
Thaw–COI–R	CTAATTAAATAGTTTAGGATTTTCT	
Fint-F1	ATTTTGACTATTACCCCCATCTC	Candidate species-specific primers targeting *F. intonsa*
Fint-R1	AAAATTTTAATTCCTGTAGGTACC	
Fint-F2	*CCATTATCAACATTCTATCATTCG*	
Fint-R2	CTTATGTTTAGTTTGGCCCCTGCG	
Fint-F3	AGGAGGGGGTGACCCAGTAC	
Fint-R3	TAGGAAAAAGTGAATTTTTAGAAG	
Focc-F1	GTTATACCAATTATAATTGGTGGG	Candidate species-specific primers targeting *F. occidentalis*
Focc-R1	CTCCTGCTAAAACTGGTAACGAC	
Focc-F2	TTGCCTGGTTTTGGTTTAATCTCC	
Focc-R2	CTGATAATTGATCCAAATCTTGAG	
Focc-F3	TTAGCAGGAGCTATTACAATATTA	
Focc-R3	GGATATCAATGTACTGTTCCTGCG	
Musi-F1	TCAGGAATTCTAGGTTTATCTCTT	Candidate species-specific primers targeting *M. usitatus*
Musi-R1	ATAAAACTGGGTCTCCTCCC	
Musi-F2	GATCTCCCATAAAACTTTTTATTC	
Musi-R2	TGAGTAATAATGTGAGAGATAAGC	
Musi-F3	TTGACTTTTACCCCCTTCAATT	
Musi-R3	TAAAATATGCTCGTGTATCAACG	
Thaw-F1	TCAACTTTTTATCATTCAGGAAAC	Candidate species-specific primers targeting *T. hawaiiensis*
Thaw-R1	GAAAATTTTAATTCCTGTTGGGACA	
Thaw-F2	CTTTTAACAGATCGAAATTTAAAC	
Thaw-R2	GTGTTGTGGAAAAAATGTTAGATT	
Thaw-F3	CTTTTAACAGATCGAAATTTAAAC	
Thaw-R3	GTGAACAAATCCTGCAAAAATTGCG	
Fint–COI–Sp-F	AGGAGGGGGTGACCCAGTAC	Final species-specific primer pair for *F. intonsa*
Fint-COI-Sp-R	TAGGAAAAAGTGAATTTTTAGAAG	
Focc-COI-Sp-F	TTAGCAGGAGCTATTACAATATTA	

Focc-COI-Sp-R	GGATATCAATGTACTGTTCCTGCG	Final species-specific primer pair for *F. occidentalis*
Musi-COI-Sp-F<br>Musi-COI-Sp-R	GATCTCCCATAAAACTTTTTATTC<br>TGAGTAATAATGTGAGAGATAAGC	Final species-specific primer pair for *M. usitatus*
Thaw-COI-Sp-F<br>Thaw-COI-Sp-R	CTTTTAACAGATCGAAATTTAAAC<br>GTGAACAAATCCTGCAAAAATTGCG	Final species-specific primer pair for *T. hawaiiensis*

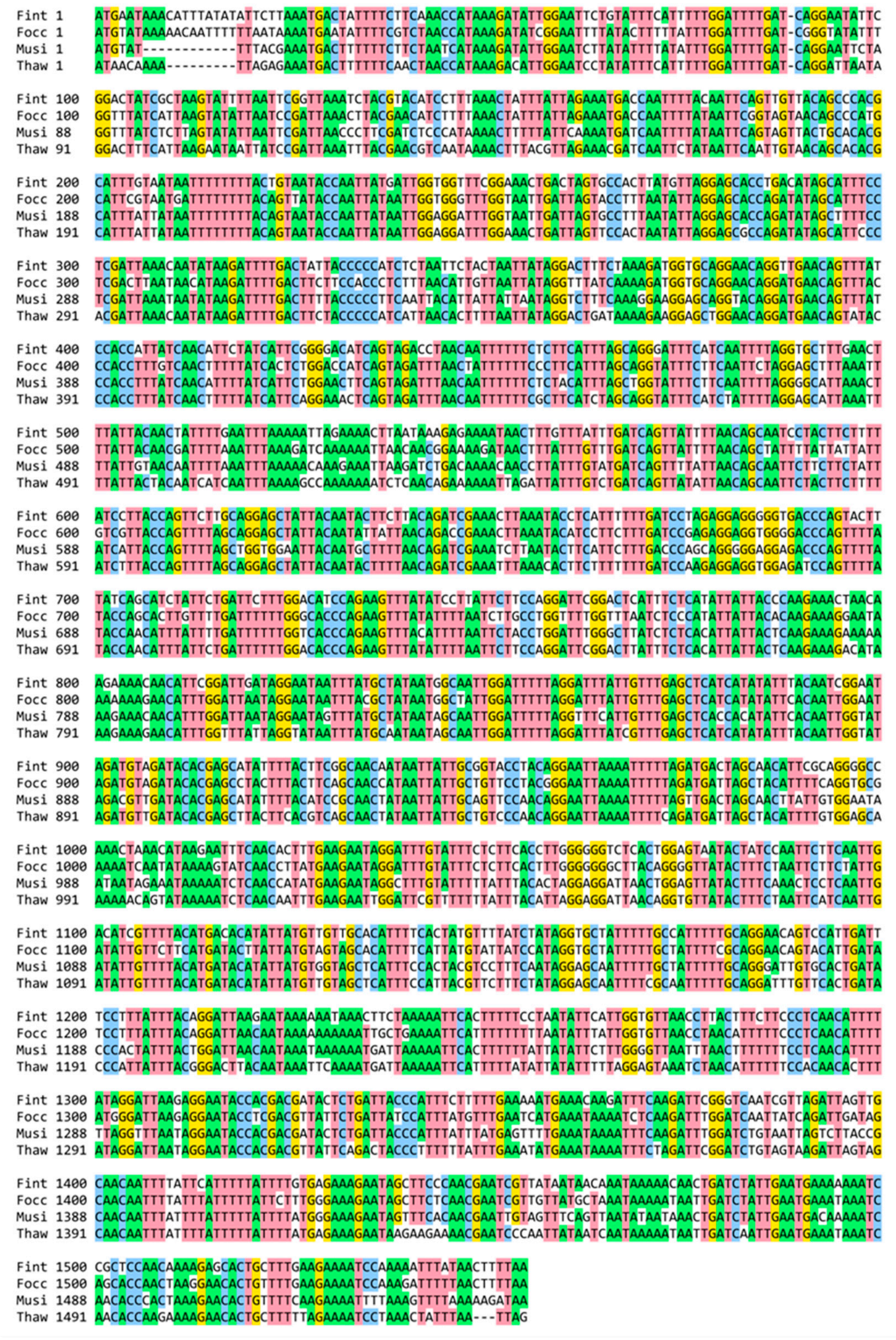

Figure S1. Sequence alignment of COI gene fragments from four thrips species Alignment of COI fragments from Fint (*F. intonsa*), Focc (*F. occidentalis*), Musi (*M. usitatus*), and Thaw (*T. hawaiiensis*), showing a polymorphism-rich region (600–1300 bp) used for species-specific primer design. Colored nucleotides denote interspecific

polymorphic sites.

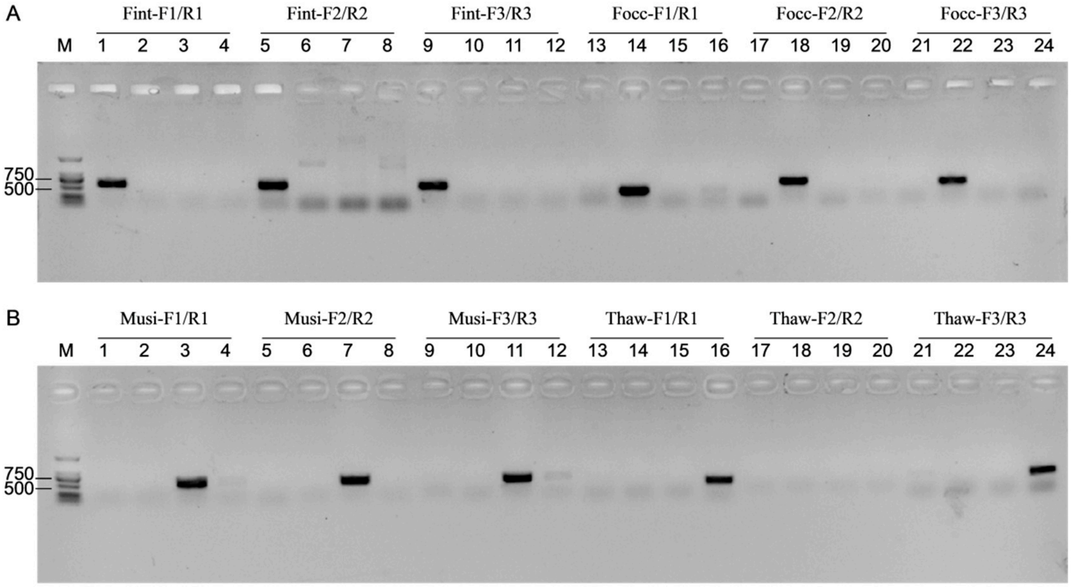

Figure S2. Primer screening using single-species DNA templates

(**A**) PCR results for candidate primers targeting Fint (*F. intonsa*) and Focc (*F. occidentalis*). Lanes 1–4: Fint-F1/R1; 5–8: Fint-F2/R2; 9–12: Fint-F3/R3; 13–16: Focc-F1/R1; 17–20: Focc-F2/R2; 21–24: Focc-F3/R3. (**B**) PCR results for candidate primers targeting Musi (*M. usitatus*) and Thaw (*T. hawaiiensis*). Lanes 1–4: Musi-F1/R1; 5–8: Musi-F2/R2; 9–12: Musi-F3/R3; 13–16: Thaw-F1/R1; 17–20: Thaw-F2/R2; 21–24: Thaw-F3/R3. The following four DNA templates were used (from lanes 1 to 4 in each group): Fint (*F. intonsa*), Focc (*F. occidentalis*), Musi (*M. usitatus*), and Thaw (*T. hawaiiensis*). M: DNA marker (DL2000).

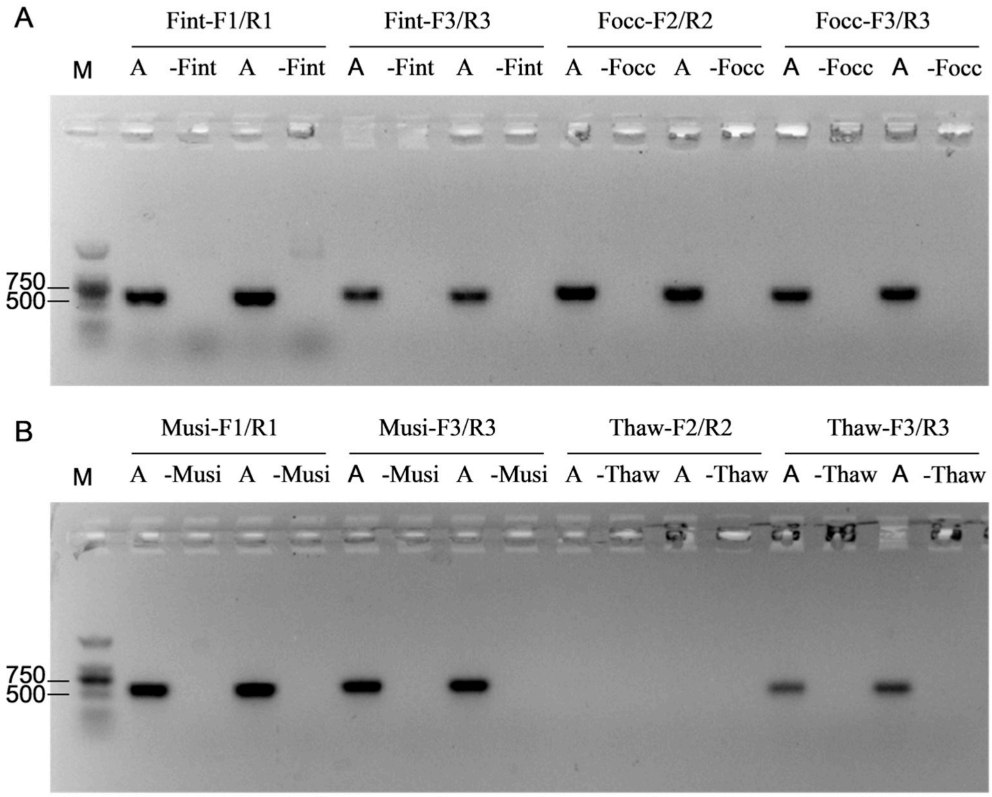

Figure S3. Validation of primer specificity under mixed-template conditions
(**A**) Primer sets for Fint (*F. intonsa*) and Focc (*F. occidentalis*) were tested using DNA mixtures. (**B**) Primer sets for Musi (*M. usitatus*) and Thaw (*T. hawaiiensis*) were tested using DNA mixtures. “A”: target species included; “-”: excluded. Distinct amplicons were observed only in “A” groups, with no amplification in “-” mixtures. M: DNA marker (DL2000).

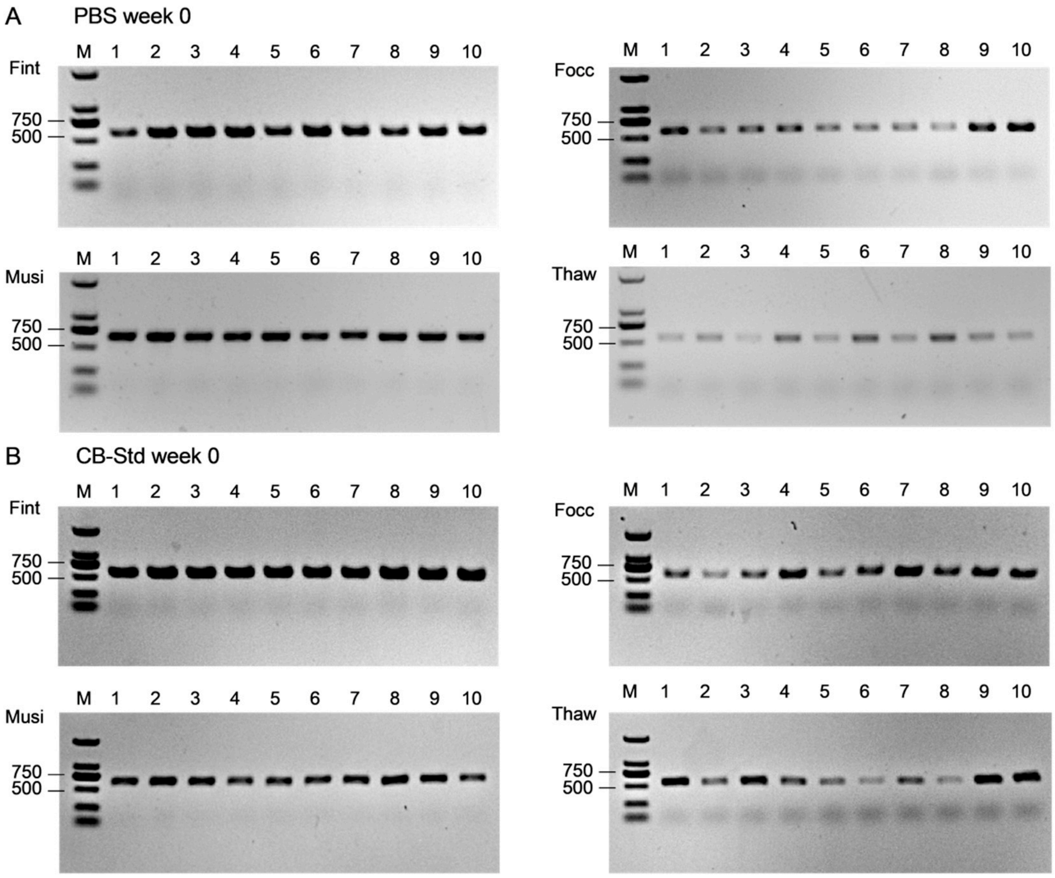

Figure S4. PCR amplification performance of PBS and CB-Std extraction methods at week 0 across four thrips species

(**A**) PBS-based extraction; (**B**) CB-Std-based extraction. Fint: *F. intonsa*; Focc: *F. occidentalis*; Musi: *M. usitatus*; Thaw: *T. hawaiiensis*. M: DNA marker (DL2000); lanes 1–10: ten biological replicates per species.

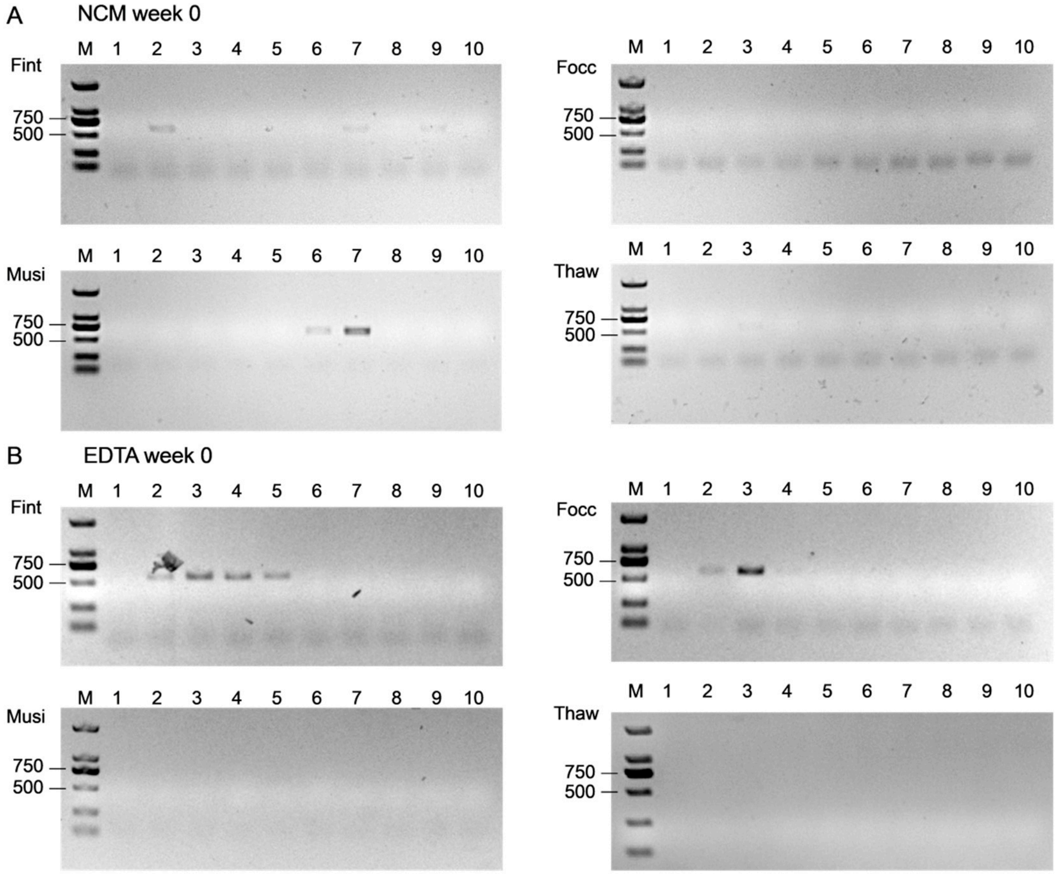

Figure S5. PCR amplification performance of NCM and EDTA extraction methods at week 0 across four thrips species

(**A**) NCM-based extraction; (**B**) EDTA-based extraction. Fint: *F. intonsa*; Focc: *F. occidentalis*; Musi: *M. usitatus*; Thaw: *T. hawaiiensis*. M: DNA marker (DL2000); lanes 1–10: ten biological replicates per species.

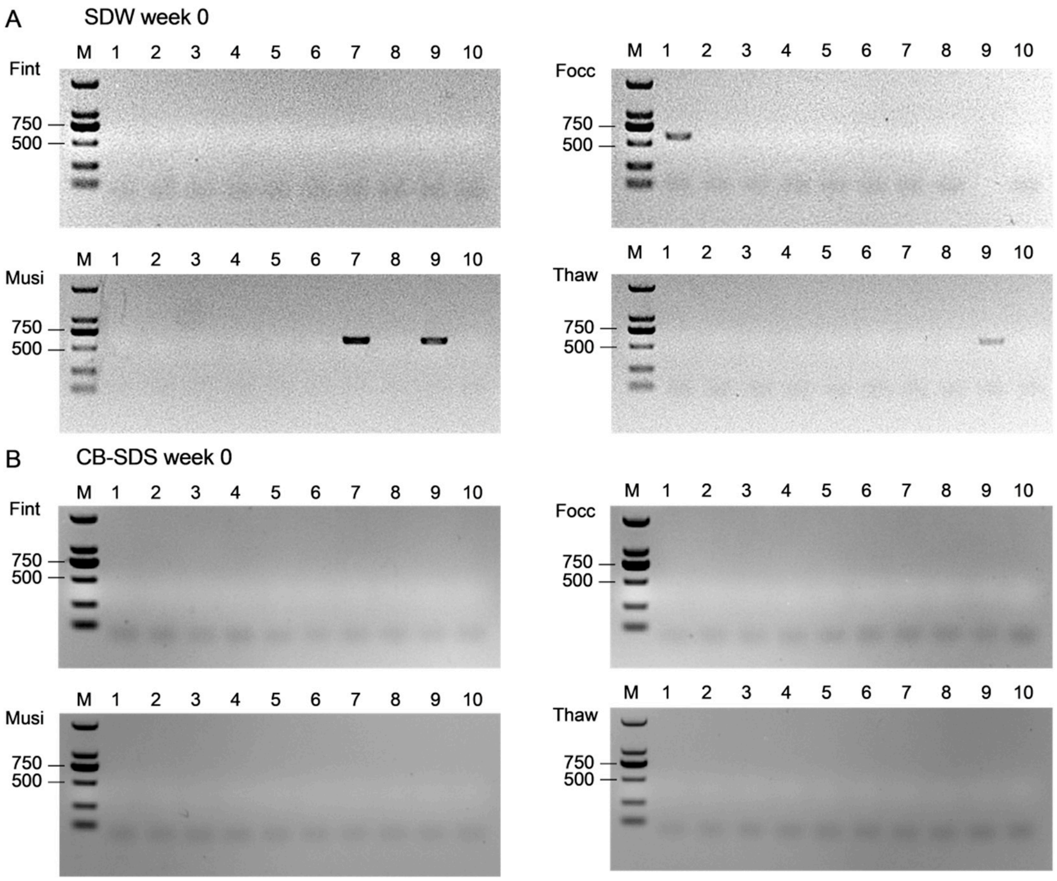

Figure S6. PCR amplification performance of SDW and CB-SDS extraction methods at week 0 across four thrips species

(**A**) SDW-based extraction; (**B**) CB-SDS-based extraction. Fint: *F. intonsa*; Focc: *F. occidentalis*; Musi: *M. usitatus*; Thaw: *T. hawaiiensis*. M: DNA marker (DL2000); lanes 1–10: ten biological replicates per species.

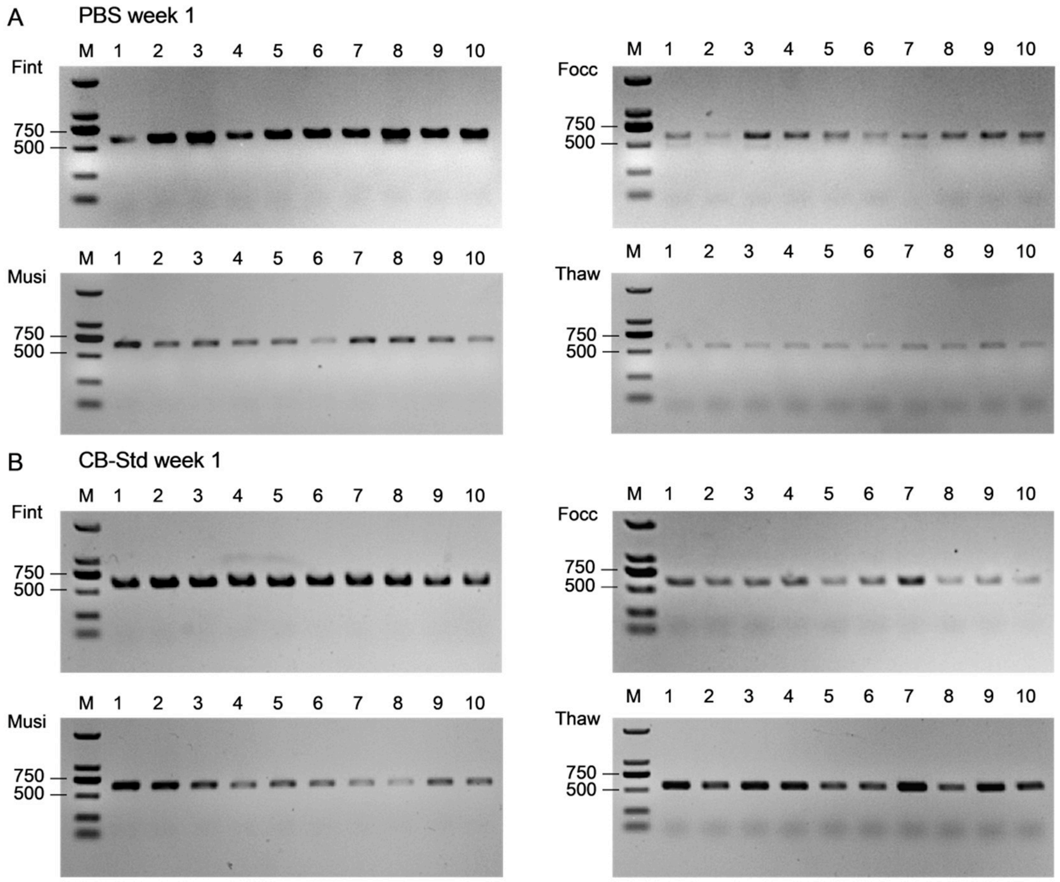

Figure S7. PCR amplification performance of PBS and CB-Std extraction methods at week 1 across four thrips species

(**A**) PBS-based extraction; (**B**) CB-Std-based extraction. Fint: *F. intonsa*; Focc: *F. occidentalis*; Musi: *M. usitatus*; Thaw: *T. hawaiiensis*. M: DNA marker (DL2000); lanes 1–10: ten biological replicates per species.

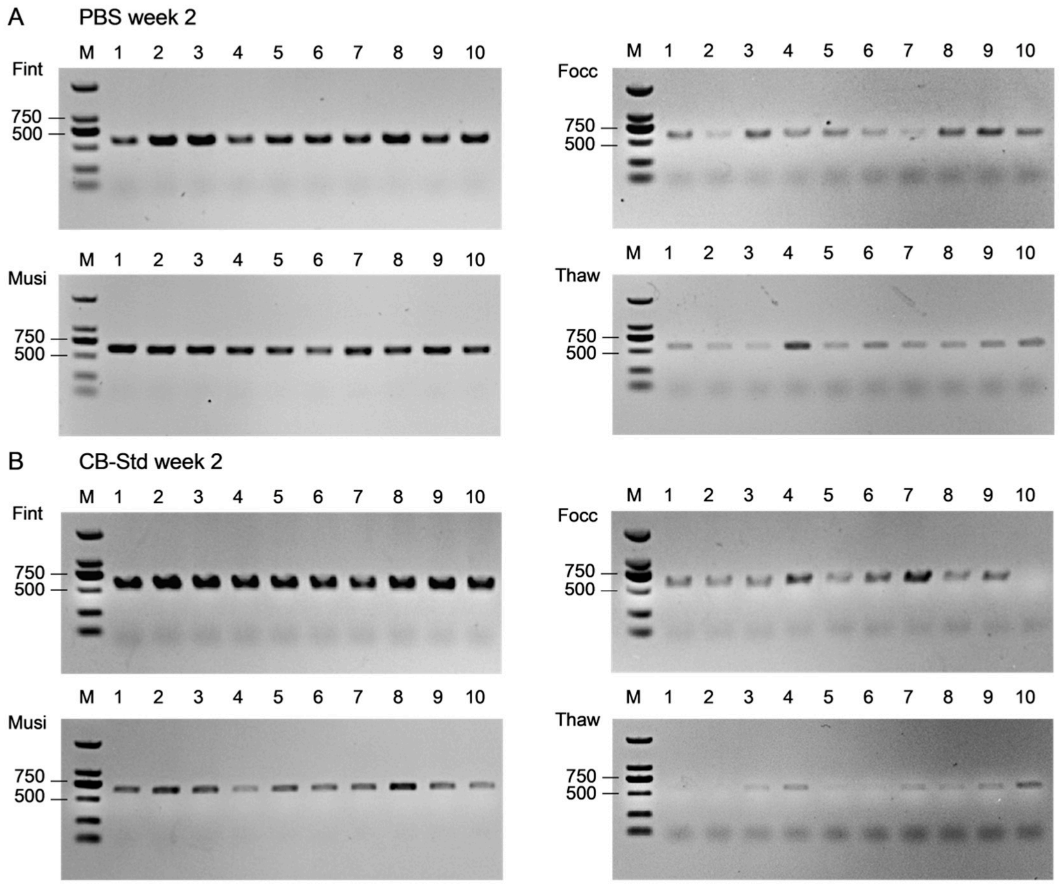

Figure S8. PCR amplification performance of PBS and CB-Std extraction methods at week 2 across four thrips species

(**A**) PBS-based extraction; (**B**) CB-Std-based extraction. Fint: *F. intonsa*; Focc: *F. occidentalis*; Musi: *M. usitatus*; Thaw: *T. hawaiiensis*. M: DNA marker (DL2000); lanes 1–10: ten biological replicates per species.

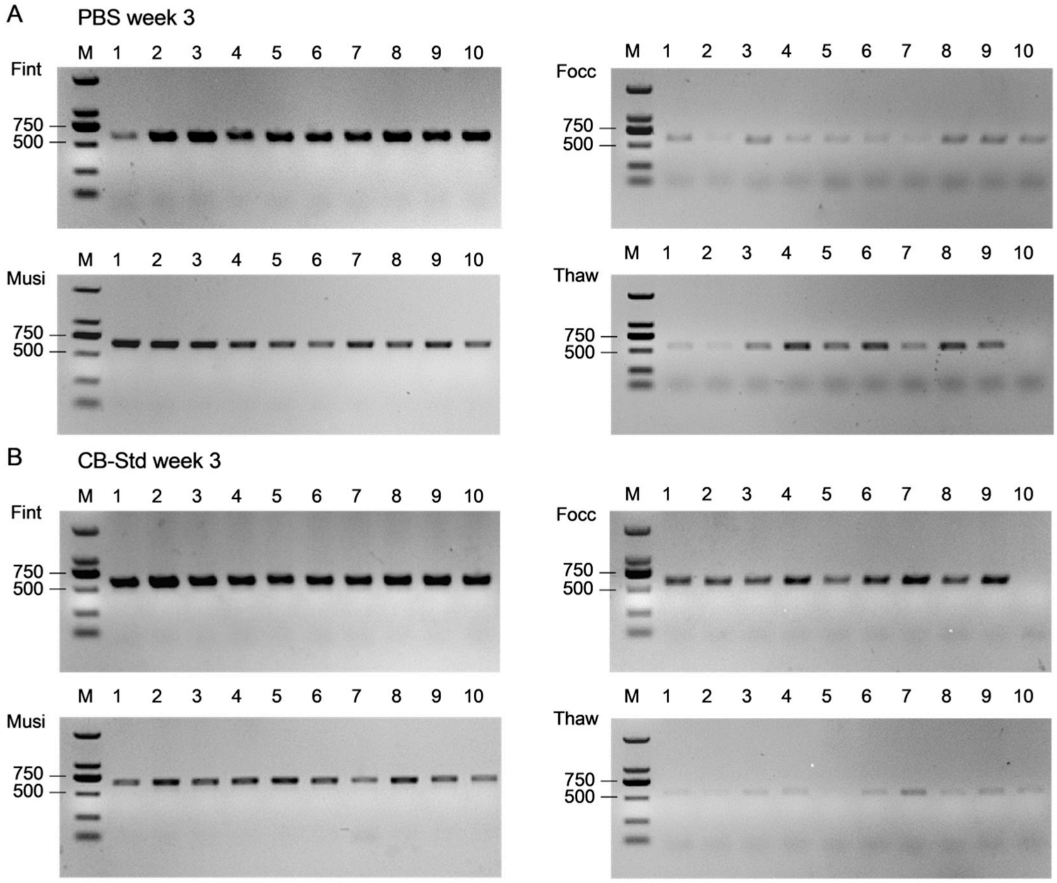

Figure S9. PCR amplification performance of PBS and CB-Std extraction methods at week 3 across four thrips species

(**A**) PBS-based extraction; (**B**) CB-Std-based extraction. Fint: *F. intonsa*; Focc: *F. occidentalis*; Musi: *M. usitatus*; Thaw: *T. hawaiiensis*. M: DNA marker (DL2000); lanes 1–10: ten biological replicates per species.

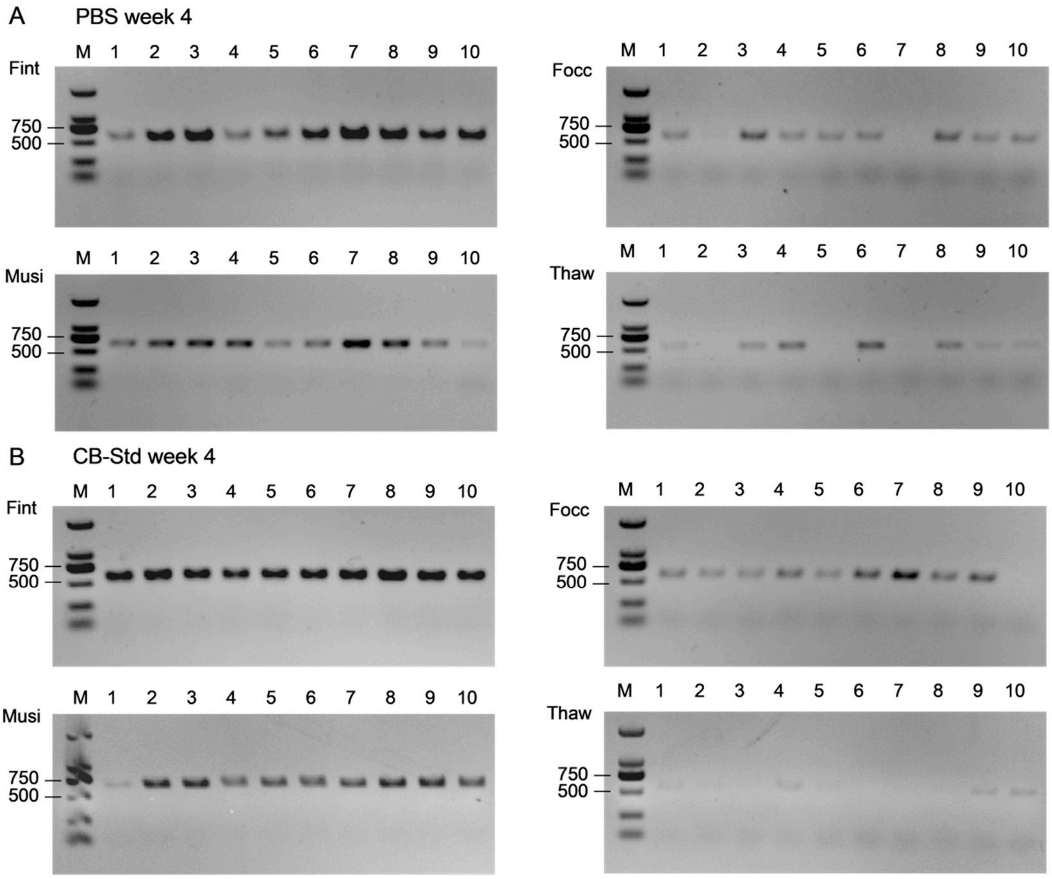

Figure S10. PCR amplification performance of PBS and CB-Std extraction methods at week 4 across four thrips species

(**A**) PBS-based extraction; (**B**) CB-Std-based extraction. Fint: *F. intonsa*; Focc: *F. occidentalis*; Musi: *M. usitatus*; Thaw: *T. hawaiiensis*. M: DNA marker (DL2000); lanes 1–10: ten biological replicates per species.

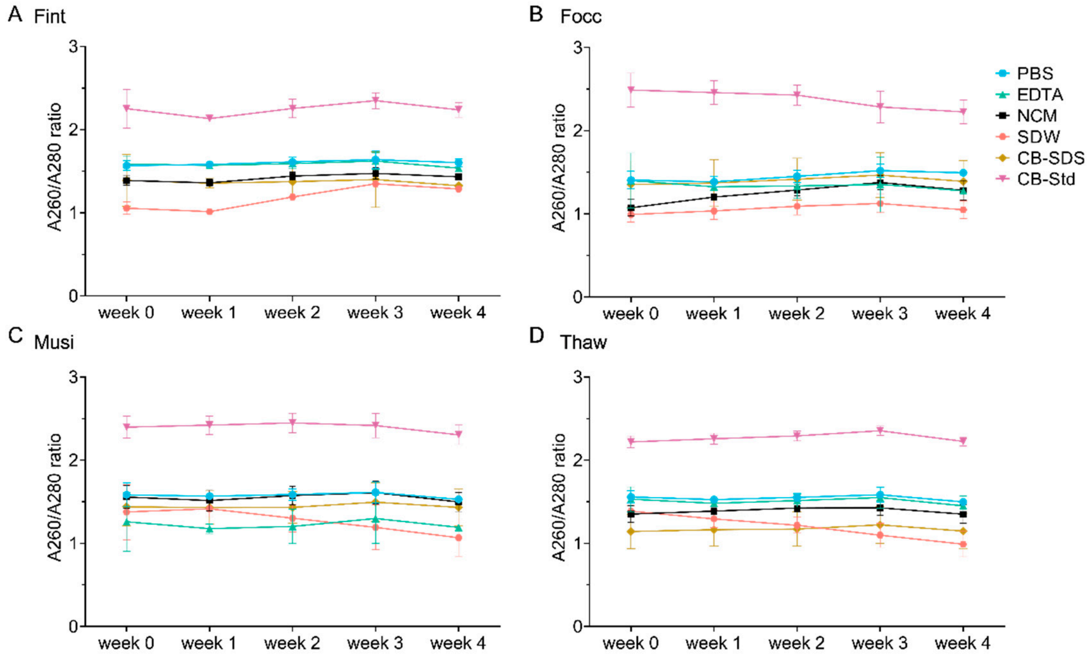

Figure S11. Temporal variation of A260/A280 absorbance ratios across extraction methods

(**A**) Fint (*F. intonsa*); (**B**) Focc (*F. occidentalis*); (**C**) Musi (*M. usitatus*); (**D**) Thaw (*T. hawaiiensis*). A260/A280 absorbance ratios were measured weekly over a four-week period to characterize temporal changes in absorbance profiles following DNA extraction using six methods: PBS, EDTA, NCM, SDW, CB-SDS, and CB-Std. Data represent the mean ± standard deviation (SD) based on ten biological replicates.

Absorbance-based ratios are shown for comparative purposes only. In detergent-free crude extracts obtained by brief heat treatment and centrifugation, these ratios reflect the relative UV absorbance of soluble components in the supernatant rather than true DNA purity and should therefore be interpreted with caution.

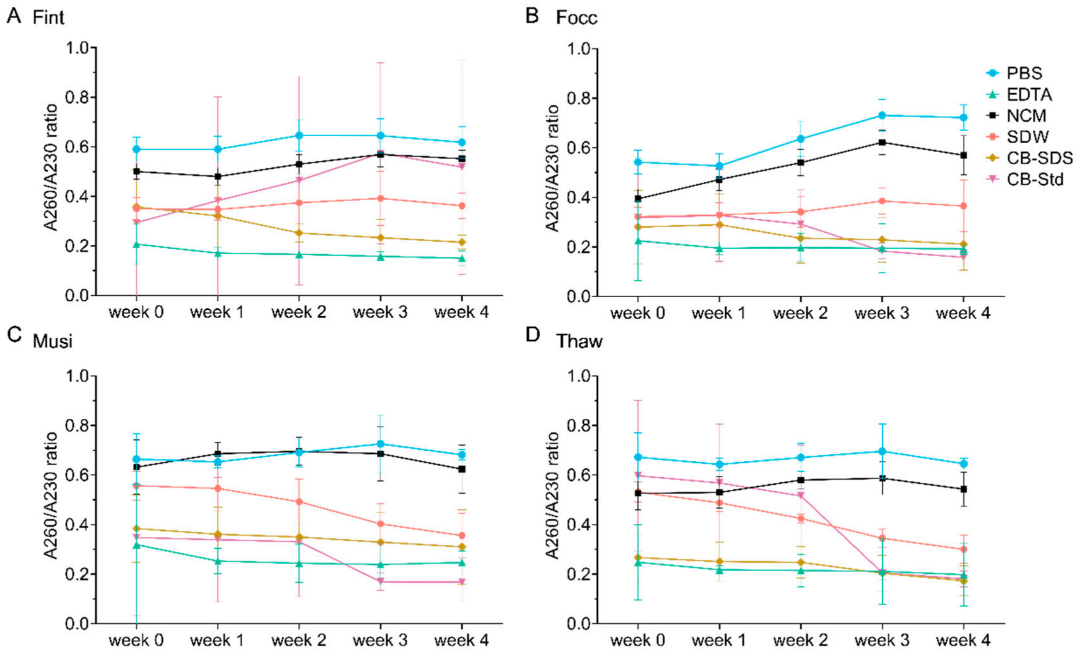

Figure S12. Temporal variation of A260/A230 absorbance ratios across extraction methods

(**A**) Fint (*F. intonsa*); (**B**) Focc (*F. occidentalis*); (**C**) Musi (*M. usitatus*); (**D**) Thaw (*T. hawaiiensis*). A260/A230 absorbance ratios were measured weekly over a four-week period to characterize temporal changes in absorbance profiles following DNA extraction using six methods: PBS, EDTA, NCM, SDW, CB-SDS, and CB-Std. Data represent the mean ± standard deviation (SD) based on ten biological replicates.

Absorbance-based ratios are shown for comparative purposes only. In detergent-free crude extracts obtained by brief heat treatment and centrifugation, these ratios reflect the relative UV absorbance of soluble components in the supernatant rather than true DNA purity and should therefore be interpreted with caution.
